# Chromone-based monoamine oxidase B inhibitor with potential iron-chelating activity for the treatment of Alzheimer’s disease

**DOI:** 10.1080/14756366.2022.2134358

**Published:** 2022-12-15

**Authors:** Changjun Zhang, Yujia Zhang, Yangjing Lv, Jianan Guo, Bianbian Gao, Yi Lu, Anjie Zang, Xi Zhu, Tao Zhou, Yuanyuan Xie

**Affiliations:** a College of Pharmaceutical Science, Zhejiang University of Technology, Hangzhou, P. R. China; b School of Food Science and Biotechnology, Zhejiang Gongshang University, Hangzhou, P. R. China; c Collaborative Innovation Centre of Yangtze River Delta Region Green Pharmaceuticals, Zhejiang University of Technology, Hangzhou, P. R. China

**Keywords:** Alzheimer’s disease, multitarget-directed ligands, MAO-B inhibitor, iron-chelating, hydroxypyridinones

## Abstract

Based on the multitarget-directed ligands (MTDLs) strategy, a series of chromone-hydroxypyridinone hybrids were designed, synthesised, and evaluated as potential multimodal anti-AD ligands. Prospective iron-chelating effects and favourable monoamine oxidase B (MAO-B) inhibitory activities were observed for most of the compounds. Pharmacological assays led to the identification of compound **17d**, which exhibited favourable iron-chelating potential (pFe^3+^ = 18.52) and selective *h*MAO-B inhibitory activity (IC_50_ = 67.02 ± 4.3 nM, SI = 11). Docking simulation showed that **17d** occupied both the substrate and the entrance cavity of MAO-B, and established several key interactions with the pocket residues. Moreover, **17d** was determined to cross the blood–brain barrier (BBB), and can significantly ameliorate scopolamine-induced cognitive impairment in AD mice. Despite its undesired pharmacokinetic property, **17d** remains a promising multifaceted agent that is worth further investigation.

## Graphical Abstract

**Figure:**
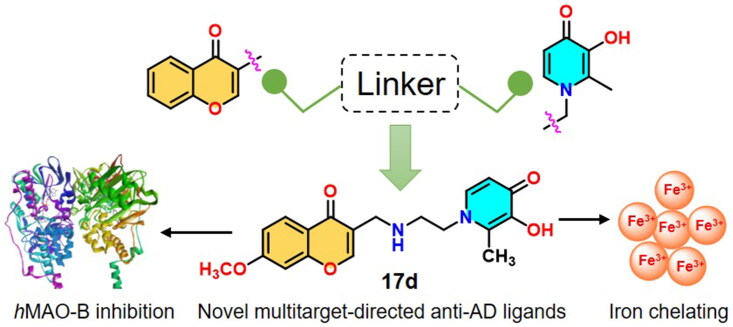

## Introduction

Disease‐modifying therapy for Alzheimer’s disease (AD) is far from satisfactory^1^. The underlying pathogenesis of AD remained frustratingly under debate despite significant efforts over the last 50 years^2^^,^^3^. Interrelated and multifaced etiopathology among AD pathological factors was proposed, especially for iron dyshomeostasis and abnormal MAO-B activities^4–6^. Elevated iron has been proved to deposit within the vulnerable neuronal populations and potentiates oxidative stress *via* the Fenton– and Haber–Weiss reactions, as well as by increasing lipid peroxidative stress^7–9^. Increased neuronal iron in AD is known to enhance Aβ production and oligomerisation, further facilitating tau dysfunction and neurofibrillary tangles^10^^,^^11^. As such, human monoamine oxidases (MAOs) are flavin adenine dinucleotide (FAD)-containing enzymes responsible for the oxidative deamination of monoamine neurotransmitters, and the elevated MAO-B activity led to a higher level of neurotoxic byproducts accelerates neurotransmitters consumption, and neuronal damage^12^^,^^13^.

**Figure 1.**
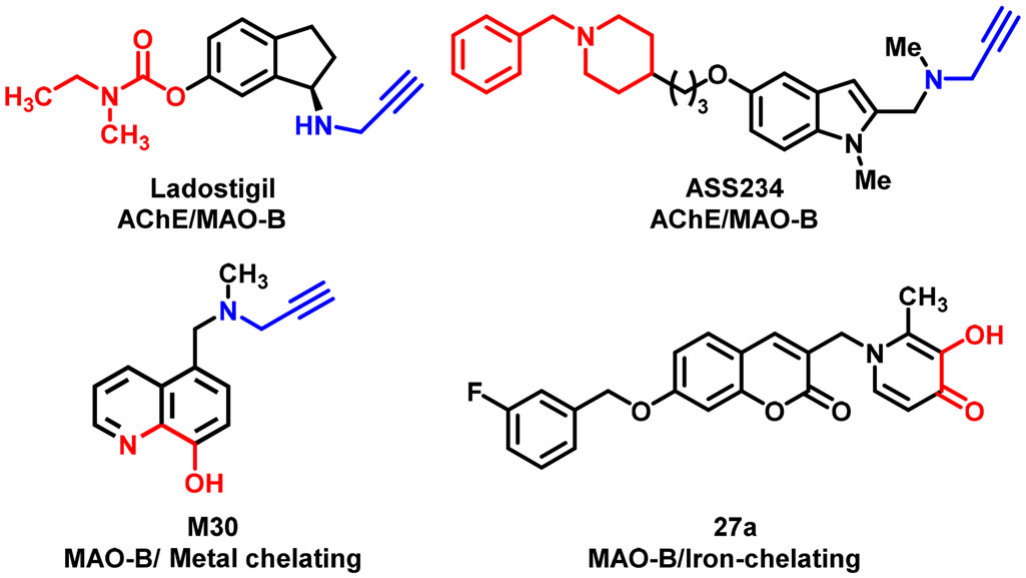
Representative dual-targeting anti-AD agents.

Recently, following the “one molecule, multiple target” paradigm, the emerged multitarget-directed ligands (MTDLs) strategy has been suggested as a powerful and promising alternative paradigm for developing effective anti-AD agents^14^^,^^15^. MAO-B has been paid increasing attention as the multifunctional anti-AD agent design target as a consequence of the neuroprotective and neurorescue effects of MAO-B inhibitors^16^. Currently, dozens of MAO-B inhibitors with auxiliary beneficial properties or target affinity (e.g. antioxidative ability, inhibit Aβ aggregation, AChE inhibition, and metal chelation) have been developed by integrating pharmacophores or scaffolds from two or more molecules and were proved to undergo unique anti-AD mechanisms (Figure 1)^17–21^.

**Figure 2.**
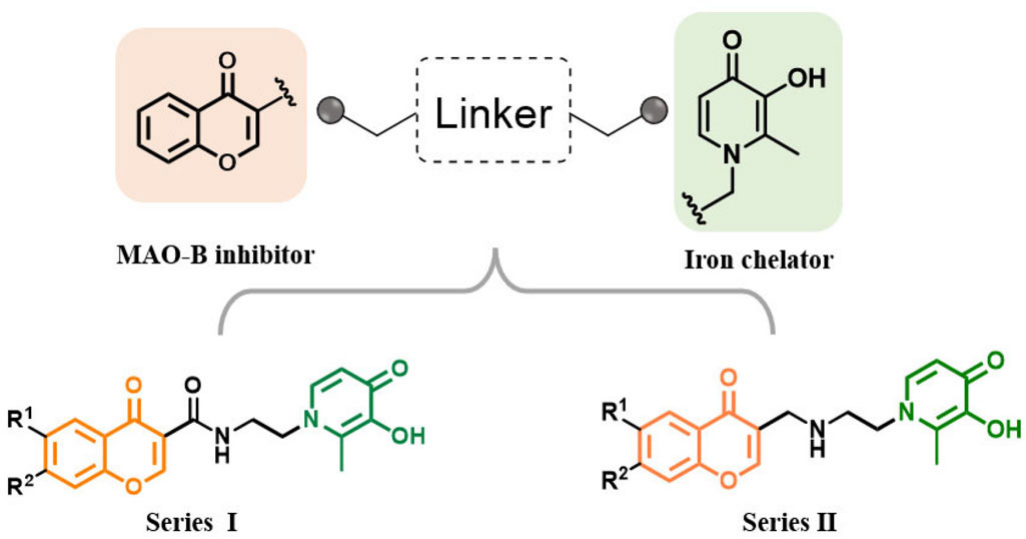
Representative dual-targeting anti-AD agents.

Several pieces of evidence reported by our research group have revealed that coumarin/benzamide-based multi-target ligands with the iron-chelating ability and MAO-B inhibitory activity have an attractive anti-AD potential^20^^,^^22^. Aiming at identifying new multipotent ligands, in this work, a new series of chromone-hydroxypyridinone hybrids were rationally designed by linking chromone core and hydroxypyridinone moiety with the appropriate linkers (Figure 2). Since studies have demonstrated that 3-carboxyl chromone is a privileged scaffold of MAO-B and possesses specific selectivity over MAO-A isoform, accordingly, the hydroxypyridinone fragment was assembled to 3-position of chromone core^23–25^. From the induce-fit perspective, the flexible ethylenediamine linker was chosen to accommodate the hydrophobic cavity as well as partially diminish the rigidity of the molecule^12^^,^^26^. Modifications on the 7-position of the chromone ring were also investigated since studies prove that benzyloxy decoration can promote selectivity and efficacy^27^^,^^28^. Thus, we synthesised and evaluated chromone-hydroxypyridinone hybrids to explore their bioactivity in association with AD.

## Results and discussion

### Chemistry

**Scheme 1.**
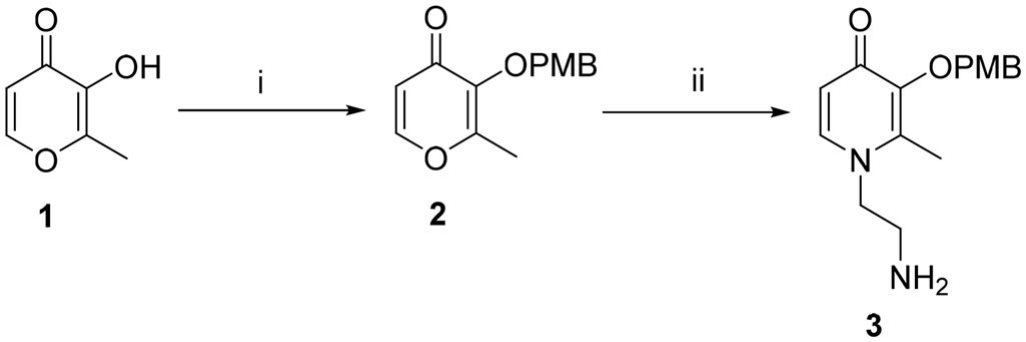
Reagents and conditions: (i) 4-Methoxybenzyl chloride, K_2_CO_3_, acetone, reflux, 3–8 h. (ii) Ethane-1,2-diamine, Ethanol: H_2_O = 1:1, 70 °C, 1.5 h.

**Scheme 2.**
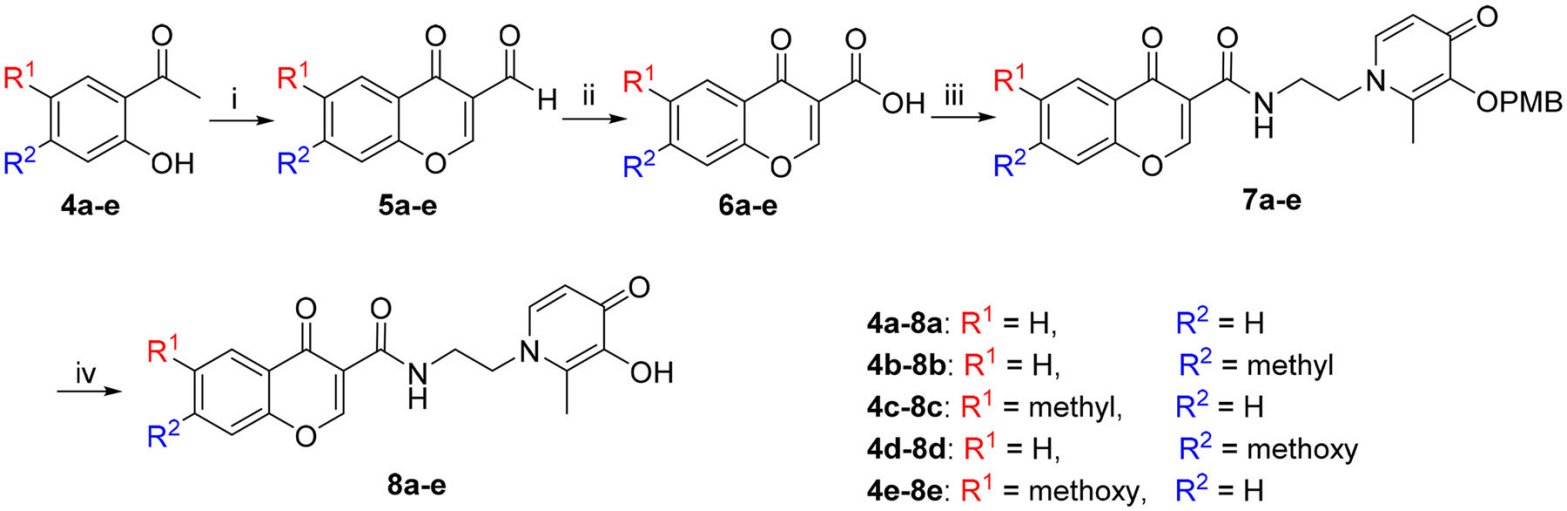
Reagents and conditions: (i) POCl_3_, DMF, −10 °C, 15 h. (ii) NH_2_SO_3_H, NaClO_2_, 0 °C, 12 h. (iii) POCl_3_, DMF, **3**, r.t., 10 h. (iv) BCl_3_, anhydrous DCM, −48 °C to r.t., 12 h.

**Scheme 3.**
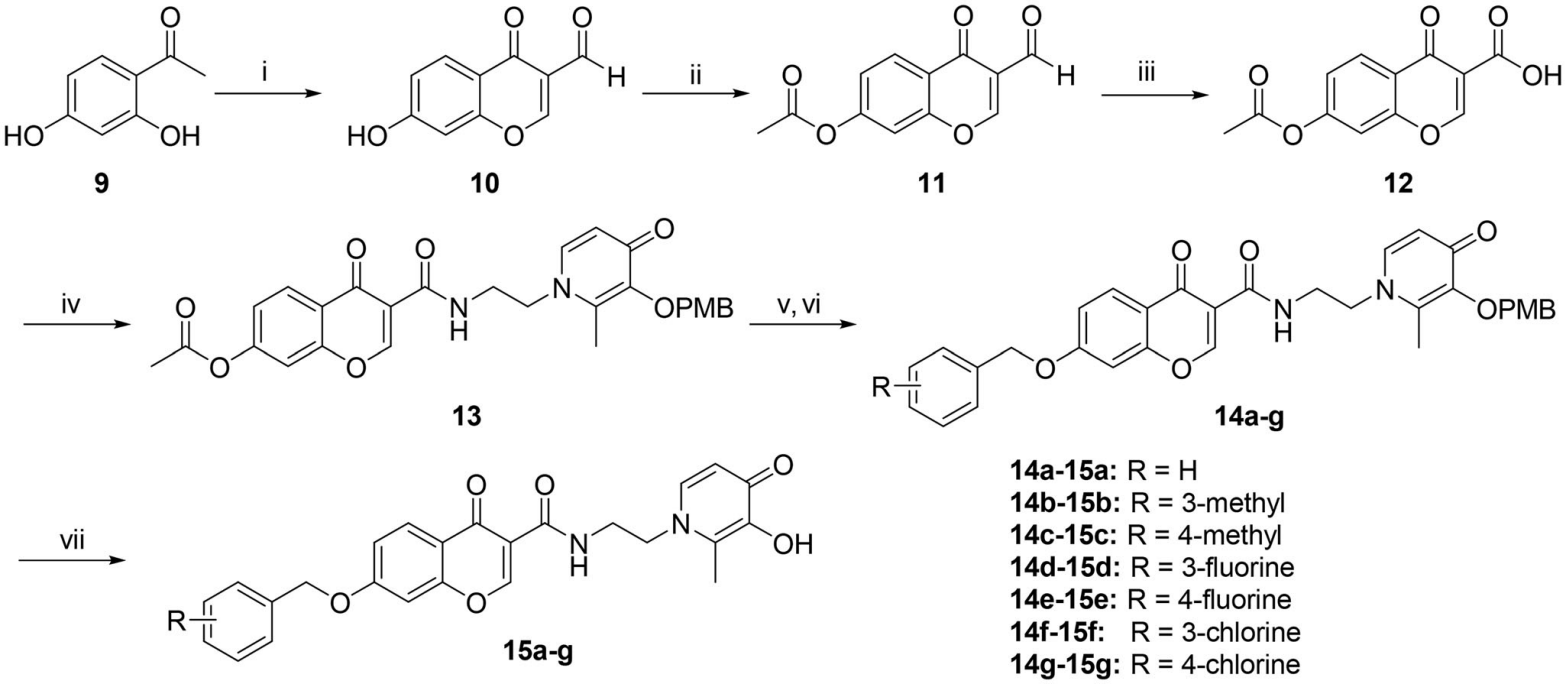
Reagents and conditions: (i) POCl_3_, DMF, −10 °C 15 h. (ii) Acetyl chloride, Triethylamine, anhydrous DCM, 0 °C to r.t., 3 h. (iii) NH_2_SO_3_H, NaClO_2_, 0 °C, 12 h. (iv) Oxalyl chloride, DMF, anhydrous DCM, **3**, r.t., 3 h. (v) K_2_CO_3_, acetonitrile: H_2_O = 1:1, reflux, 0.5 h. (vi) corresponding substituted benzyl bromide, K_2_CO_3_, DMF, 12 h (vi) BCl_3_, anhydrous DCM, −48 °C to r.t., 12 h.

According to the difference in linkers, two series of chromone derivatives were outlined: (I) amide-bonded compounds, and (II) C–-N bonded compounds. The amide-bonded chromone derivatives (**8a–e**, **15a–g**) were efficiently obtained according to the procedure shown in Schemes 1–3. Intermediate **2** was synthesised starting from commercially available maltol through the protection of the 3-hydroxyl group. Subsequently, the protected maltol was converted into intermediate **3** by reaction with ethylenediamine under reflux conditions (Scheme 1).

Appropriate chromone-carbaldehydes (**5a–e**) were synthesised by corresponding acetophenone through the Vilsmeier–Haack reaction^23^. Afterward, the chromone carboxylic acids (**6a-e**) were prepared by Pinnick oxidation, in the presence of sodium chlorite^29^. The chromone-carboxamide compounds (**7a–e**) were obtained through a reaction that included the generation of an acyl chloride intermediate and the subsequent addition of intermediate **3** (Scheme 2).

The synthetic pathway followed to obtain the first series of compounds (**15a–g**) involved seven steps (Scheme 3). Except for the difference in acyl chloride reagents, the synthetic procedure of compound **13** was the same as that of the chromone-carboxamide derivatives (**7a–e**). Subsequently, the ester group of compound **13** was hydrolysed with potassium carbonate and treated with appropriate benzyl bromide to afford the compounds (**14a–g**).

**Scheme 4.**
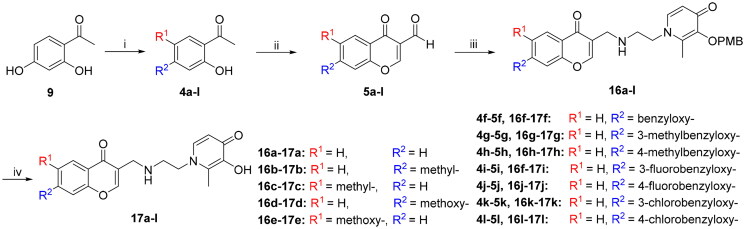
Reagents and conditions: (i) 4-Methoxybenzyl chloride, K_2_CO_3_, acetone, reflux, 3–8 h. (ii) Ethane-1,2-diamine, Ethanol: H_2_O = 1:1, 70 °C, 1.5 h.

In turn, the second series of compounds (**16a–l**) were synthesised by reductive amination, starting from chromone-carbaldehydes (**5a–l**) to yield the imine intermediate (Scheme 4). Finally, the chromone-based compounds (**8a–e, 15a–g**, and **16a–l**) were performed by a selective debenzylation reaction with boron trichloride to remove the 4-methoxybenzyl protecting groups.

### Iron-chelating ability test

Recently, iron has been determined as a pro-oxidant involved in oxidative damage due to Fenton– and Haber–Weiss reactions and ferroptosis^30^. To evaluate the iron chelating capacity of chromone derivatives, all of the compounds were then subjected to determine the p*K*_a_ values and iron affinity constants for iron (III) by an automated spectrophotometric titration system^20^. The obtained data are provided in Table 1. All derivatives displayed impressive pFe^3+^ values ranging from 15.87 to 19.08 (Table 1). Compound **8e** revealed the most potent iron-chelating ability (pFe^3+^ = 19.08). Overall, no discernible difference in iron-chelating activity exists between the two series of compounds (compounds **8a–e**, **15a–g**, and **17a–l)**, both of which are outstanding.

**Table 1.** Physicochemical parameters for compounds **8a–e**, **15a–g**, and **17a–l**.

Comp.	p*K*_a1_^a^	p*K*_a2_^a^	Log *β*_1_^c^	Log *β*_2_^c^	Log *β*_3_^c^	pFe^3^^+^
**8a**	3.29	9.67	13.67	25.22	34.95	18.68
**8b**	3.32	9.58	13.74	25.04	34.36	18.35
**8c**	3.26	9.69	13.86	25.07	33.48	17.19
**8d**	3.20	9.60	13.82	25.31	34.23	18.18
**8e**	3.18	9.67	13.67	25.38	35.37	19.08
**15a**	3.30	9.59	13.77	24.72	32.17	16.45
**15b**	3.06	9.73	13.64	25.57	34.21	17.80
**15c**	3.19	9.34	13.40	23.16	31.01	15.87
**15d**	3.36	9.52	13.50	25.31	34.85	19.01
**15e**	2.89	9.57	13.24	24.65	34.50	18.52
**15f**	3.35	9.52	13.79	25.03	33.91	18.09
**15g**	3.25	9.56	13.82	24.02	31.70	16.00
**17a**	2.81	9.80	13.46	25.19	35.28	18.62
**17b**	2.95	9.82	13.58	25.38	35.34	18.61
**17c**	2.75	9.76	13.31	25.00	34.07	17.55
**17d**	2.86	9.78	13.50	25.21	35.13	18.52
**17e**	2.76	9.83	13.32	25.08	34.89	18.12
**17f**	2.73	9.69	13.20	24.70	33.77	17.45
**17g**	2.68	9.62	13.02	24.75	33.78	17.66
**17h**	2.56	9.59	12.87	24.73	33.36	17.34
**17i**	2.58	9.68	12.93	24.99	34.10	17.81
**17j**	2.76	9.53	13.13	24.78	33.73	17.89
**17k**	2.53	9.43	12.99	24.76	33.88	18.31
**17l**	2.61	9.47	13.02	24.99	34.39	18.70
**DFP**	3.65	9.78	13.96	26.43	36.36	19.75
**DFP** ^b^	3.61	9.78	15.03	27.42	37.35	20.74
**DFP** ^a^	3.65	9.78	14.67	27.58	36.90	20.29

^a^The compounds were measured in 0.1 M KCl.

^b^The data from the reference tested in a 0.1 M KCl solution^31^.

^c^The compounds were tested in DMSO: KCl (0.1 M) = 1:1.5 (*v/v*) to address the solubility issue.

**Figure 3.**
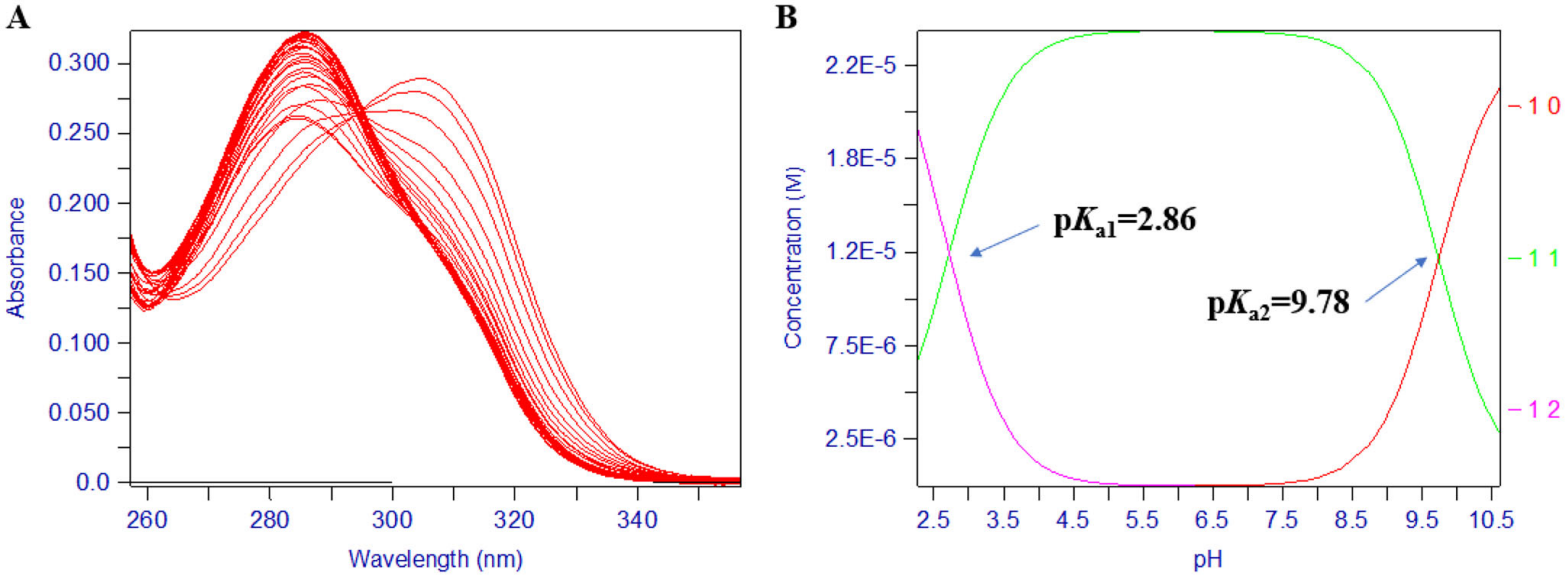
Rational design of chromone-pyridinone hybrids as potential anti-AD agents.

**Figure 4.**
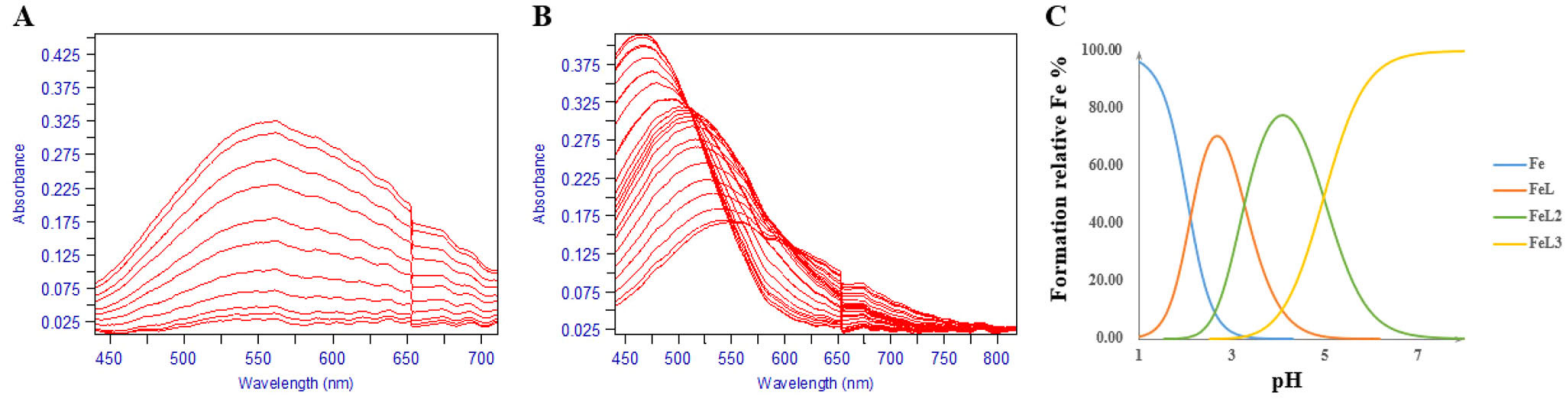
The pH-dependent UV spectra of compound **17d**. (A) The pH-dependence of the spectrum of compound **17d** in the presence of Fe^3+^ over the pH range 0.8–2.1 in 0.1 M KCl at 25 °C, [Fe^3+^] = 1.0 μM, [**17d**] = 1.1 μM. (B) The pH-dependence of the spectrum of compound **17d** in the presence of Fe^3+^ over the pH range 2.1 and 9.0 in 0.1 M KCl: DMSO = 3:2 (*v/v*) at 25 °C, [Fe^3+^] = 1.0 μM, [**17d**] = 5.0 μM. **C.** Speciation plot of Fe^3+^/**17d** as measured by the percentage formation relative to [Fe^3+^]_total_ as a function of pH. This plot was calculated from the affinity constants reported in Table 1 and the Fe^3+^ hydrolysis constants were as follows: FeOH = –2.563, Fe (OH)_2_ = –6.205, Fe (OH)_3_ = –15.100, Fe_2_ (OH)_2_ = –2.843, Fe_3_ (OH)_4_ = –6.059, and Fe (OH)_4_ = –21.883.

**Figure 5.**
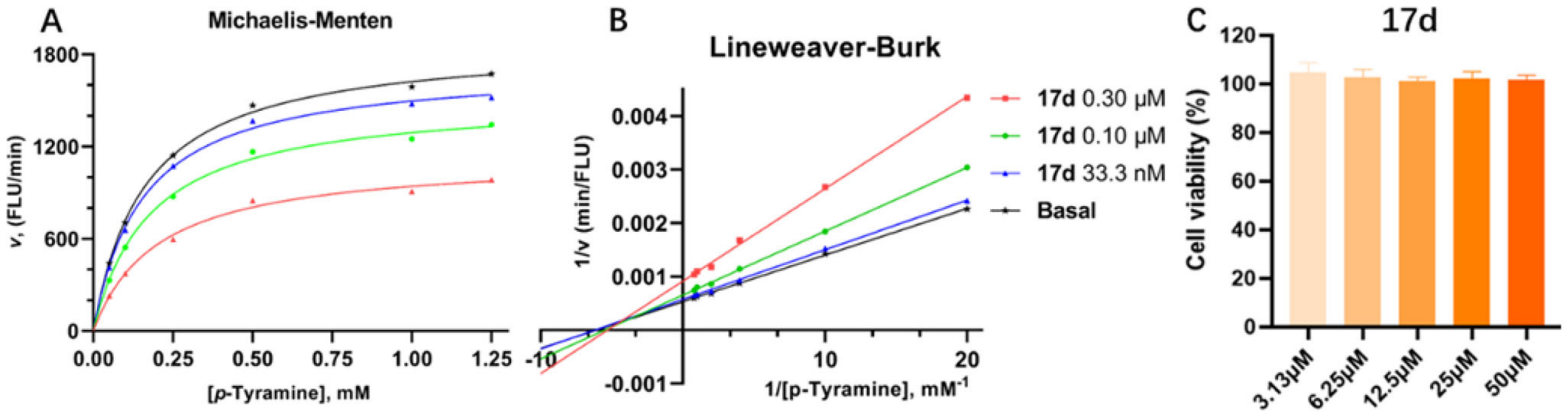
Kinetic study and cytotoxicity assay of compound **17d**. Mode of MAO-B inhibition saturation curves (A) and Lineweaver − Burk plot (B) of the inhibition of MAO-B enzyme by different concentrations of **17d** (0, 33.3, 100, and 300 nM) in the presence of *p*-tyramine (0.05, 0.1, 0.25, 0.5, 1.0, and 1.25 mM) as a substrate. The Cytotoxicity effect of compound **17d** on PC-12 cells (C).

Typically, 3-hydroxypyridin4-one derivatives are bidentate ligands (3:1 complex) and their proton ionisation characteristics, the UV spectrums yielded two pKa values (p*K*_a1_ < 3.40, p*K*_a2_ = 9.32 − 10.05). The 2D UV titration profile of **17d** over the pH range 1.3–11.0 (Figure 3(A)) yielded two pKa Values, 2.86 and 9.78 demonstrating the pH dependence of the ligand ionisation equilibrium (Figure 4(B)). Repeated the titration in the presence of iron (III), over the pH range of 2.1 − 9.0, the visible spectrum reveals three species, FeL, FeL_2_, and FeL_3_, respectively, in the solution system. The spectral resolution of these three substances led to the determination of the corresponding three equilibrium constants (compound **17d**): Log *β*_1_ (13.50), Log *β*_2_ (25.21) (Figure 5(A)), and Log *β*_3_ (35.13) (Figure 5(B)), which were the basis for calculating the final pFe^3+^ value. The abundance of each species varied with pH as illustrated in Figure 5(C).

As shown in Table 1, all of the compounds well inherited the iron-chelating ability of the hydroxypyridinone pharmacophore. Compounds with 6,7-alkyl and 6,7-alkoxy substitutions on the benzene ring showed better iron chelation than benzyloxy substitutions. Meanwhile, amide bonds linked compounds exhibited better iron chelation than C–N bond-linked derivatives.

### HMAO-B/a inhibition assay and selectivity

Subsequently, the MAOs inhibitory activity of the compounds was measured, as shown in Tables 2 and 3. As detailed below, generally, hybrids with C–N bond linkers were more potent than those amide bond-linked compounds, with most of them having inhibition rates over 40%. Chromones bearing substituents in position C-7 of chromone exhibited better MAO-B inhibitory activity with IC_50_ values ranging from micromolar concentration to nanomolar concentration. Furthermore, when the benzyloxy group was inserted into the C-7 of chromone, the inclusion of electron-withdrawing substituents (F, Cl) in the benzyloxy counterposition demonstrated higher activity than interposition replacement. The most promising chromone derivatives, on the other hand, are changed by alkyl groups at C-6 or C-7 of chromone nuclear (compounds **17a–e** and IC_50_ values range from 67 to 88 nM). Among them, compound **17d** showed the best inhibitory activity compared with the reference drug (Pargyline).

**Table 2.** The *h*MAO-B inhibitory activities of compounds.

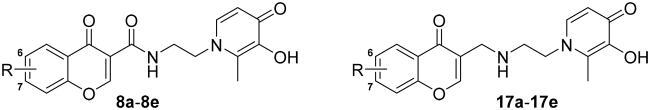					
Comp.	R	Inhibitory rate (%, 100 nM)	Comp.	R	Inhibitory rate (%, 100 nM)
**8a**	H-	42.20 ± 3.7	**17a**	H-	53.11 ± 1.6
**8b**	7-methyl-	35.79 ± 0.2	**17b**	7-methyl-	49.50 ± 0.3
**8c**	6-methyl-	42.10 ± 0.8	**17c**	6-methyl-	44.23 ± 0.1
**8d**	7-methoxy-	41.87 ± 1.4	**17d**	7-methoxy-	48.88 ± 1.4
**8e**	6-methoxy-	34.28 ± 2.2	**17e**	6-methoxy-	46.40 ± 1.3
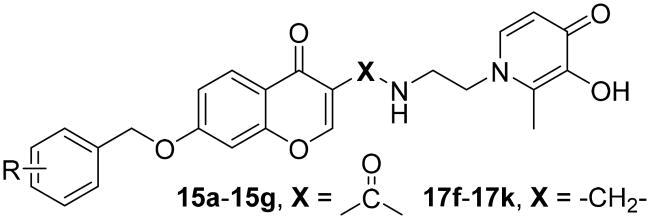					
**15a**	H-	38.95 ± 1.1	**17f**	H-	49.00 ± 0.6
**15b**	3-methyl-	39.13 ± 1.1	**17g**	3-methyl-	55.75 ± 2.7
**15c**	4-methyl-	38.70 ± 0.5	**17h**	4-methyl-	30.10 ± 3.7
**15d**	3-fluoro-	38.48 ± 1.4	**17i**	3-fluoro-	39.48 ± 0.3
**15e**	4-fluoro-	42.41 ± 2.1	**17j**	4-fluoro-	45.95 ± 0.7
**15f**	3-chloro-	41.19 ± 2.8	**17k**	3-chloro-	43.06 ± 2.0
**15 g**	4-chloro-	39.07 ± 0.5	**17l**	4-chloro-	61.43 ± 3.4
Pargyline		48.93 ± 1.7			

**Table 3.** IC_50_ and SI for the chromone derivatives on the enzyme activity of MAO isoforms.

Comp.	IC_50_ (nM)		
	*h*MAO-B	*h*MAO-A	SI^a^
**8a**	112.00 ± 1.3	–^b^	>89
**8c**	111.75 ± 5.7	**–** ^b^	>89
**8d**	107.25 ± 5.6	**–** ^b^	>93
**15e**	106.35 ± 0.2	**–** ^b^	>94
**15f**	129.50 ± 0.8	**–** ^b^	>80
**17a**	67.27 ± 1.9	2063 ± 35.4	30.7
**17b**	82.78 ± 0.7	**–** ^b^	>120
**17c**	86.30 ± 0.7	**–** ^b^	>116
**17d**	67.02 ± 4.3	739 ± 21.9	11.0
**17e**	88.66 ± 5.3	**–** ^b^	>112
**17f**	76.45 ± 3.8	795 ± 0.7	10.4
**17g**	78.00 ± 4.3	1666 ± 5.7	21.4
**17j**	101.66 ± 3.2	**–** ^b^	>99
**17k**	113.90 ± 3.8	**–** ^b^	>88
**17l**	75.56 ± 3.1	1312 ± 13.4	17.4
Pargyline	111.30 ± 0.6	4184 ± 7.1	37.6

^a^SI: *h*MAO-B selectivity index = IC_50_ (*h*MAO-A)/IC_50_ (*h*MAO-B). ^b^Inhibitory rate less than 20% at 10 μM (highest concentration tested).

Further, the inhibitory activity of compounds against *h*MAO-A isoform was tested and calculated as a selectivity index [Selectivity index (SI): IC_50_ (*h*MAO-A)/IC_50_ (*h*MAO-B)]. As indicated in Table 3, all compounds demonstrated varying degrees of selectivity, with compound **17b** exhibiting high selectivity for MAO-B. Comprehensively considering the iron-chelating ability and the inhibitory activity over enzyme isoforms, compounds **17a** and **17d** could be performed in subsequent assays.

### PAMPA-BBB assay

Good blood–brain barrier (BBB) permeability is the key to exerting drug efficacy in the treatment of central nervous system diseases. To evaluate whether the target compound could penetrate BBB, a parallel artificial membrane permeability assay (PAMPA) was used to predict^32^^,^^33^. As outlined in Table 4, The *P*_e_ value of compound **17a** measured by this method was 1.22 ± 0.22 × 1 0^−6 ^cm/s (CNS-), indicating that compound **17a** had poor permeability and could not penetrate the BBB. However, the *P*_e_ value of compound **17d** was between 2 and 4, suggesting that compound **17d** had the possibility of penetrating the BBB. To further confirm these results, a prediction platform (ADMETlab) was selected for verification. Both compounds displayed the desired CNS drug-like properties (MW ≤ 450, HBD ≤ 5, HBA ≤ 10, and Log *p* ≤ 5) and were predicted to pass through the BBB, which is classified as BBB+. Therefore, due to its potential ability to cross the BBB, compound **17d** was selected for subsequent assay.

**Table 4.** Predicted drug-like properties and PAMPA-BBB results for **17a** and **17d**.

Compound	MW^b^	HBD^b^	HBA^b^	LogP^b^	*P*_e_ ± SEM (*10^−6^ cm/s)^a^	CNS^c^	BBB permeability^b^
**17a**	326.35	2	6	1.76	1.22 ± 0.22	–	+
**17d**	356.38	2	7	1.77	2.73 ± 0.07	±	+

^a^Data are the mean ± SEM of eight independent experiments.

^b^These results were predicted *via* online programs (https://admet.scbdd.com).

^c^CNS (+) (high BBB permeation predicted); *P*_e_ (× 10^−6 ^cm s^− 1^) > 4.0; CNS (±) (BBB permeation uncertain); *P*_e_ (× 10^−6 ^cm s^−1^) from 2.0 to 4.0; CNS (−) (low BBB permeation predicted); *P*_e_ (× 10^−6 ^cm s^−1^) <2.0.

### Kinetics study and cytotoxicity assay of compound 17d

To examine the interaction mode of **17d** on MAO-B, kinetic studies were carried out. From the Lineweaver–Burk plots (Figure 5(B)), we observed that **17d** operated by a non-competitive inhibition mechanism. Using GraphPad Prism, the Michaelis constant (*K*_m_), the maximal velocity (*V*_max_) and the inhibition constant (*K*_i_) were calculated (*K*_m_ = 117.7 μM, *V*_max_ = 1701, and *K*_i_ = 74.71 nM). Subsequently, the potential toxic effect of **17d** was investigated on pheochromocytoma (PC-12) cells. Compound **17d** exhibited no cytotoxicity even at the concentration of 50 µM (Figure 5(C)).

**Figure 6.**
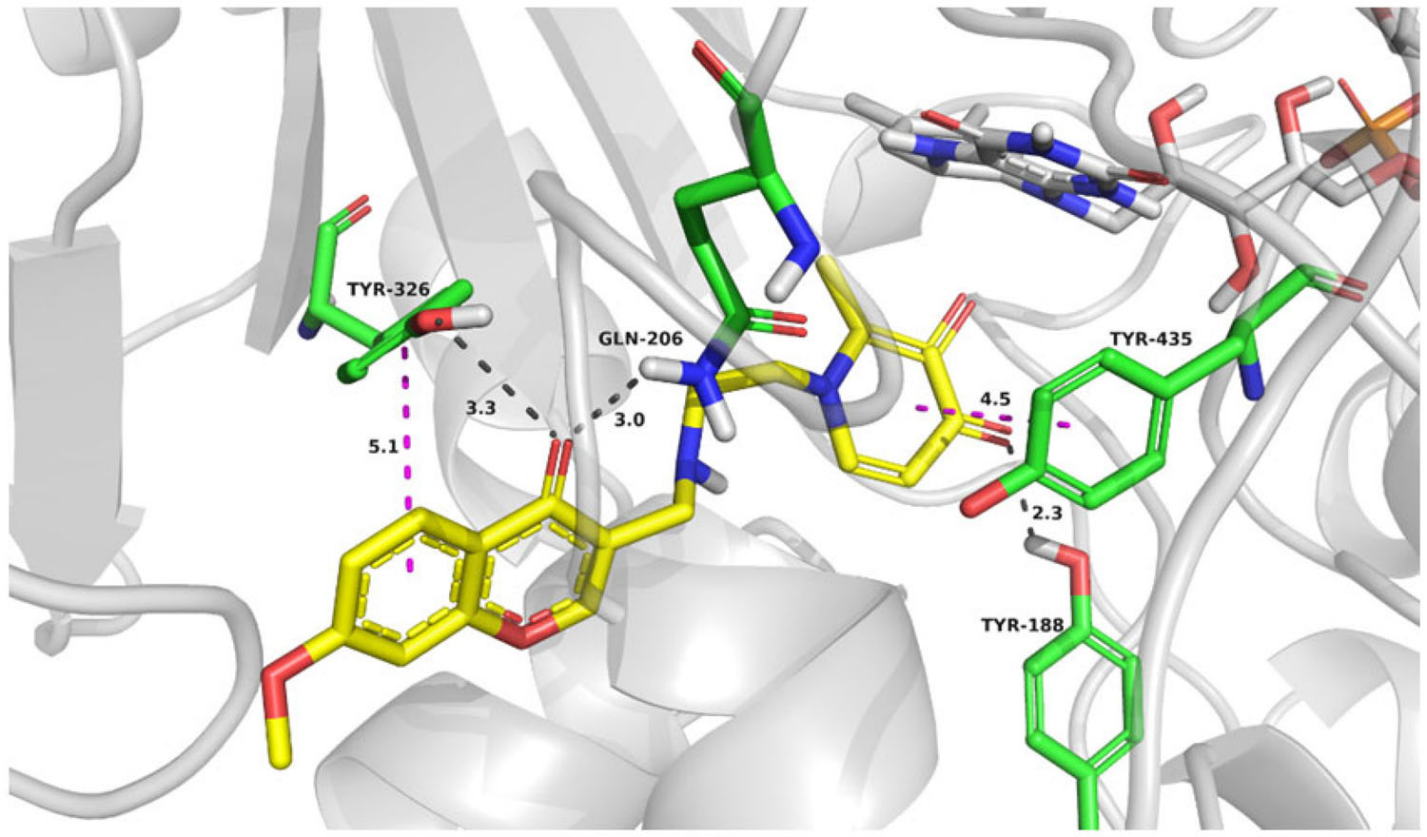
Best docking poses of compound **17d** at the active site of MAO-B. The most relevant interacting residues are presented in green carbons polytube, FAD cofactor is shown as grey carbons polytube and ligands as yellow carbons polytube.

### Molecular modelling

The potential binding conformation of compound **17d** in MAO-B (PDB: 2V5Z) was investigated by molecular docking simulation in Discovery studio version 4.0 (BIOVIA, USA). As shown in Figure 6, **17d** occupied both the entrance and substrate cavities of MAO-B with the 7-methoxy-chromone ring oriented towards the highly hydrophobic entrance cavity and formed Pi–Pi interaction with TYR 326. Moreover, the chromone-carbonyl oxygen established two hydrogen bonds with residues TYR 326 (3.3 Å) and GLN 206 (3.0 Å). Accordingly, The pyridinone moiety of **17d** occupied the substrate cavity in front of the FAD cofactor. Pi–Pi interaction was observed between the pyridinone plane and TYR 435, and a hydrogen bond interaction was monitored between pyridinone-carbonyl oxygen and the residue TYR 188. The above binding mode indicated significant interactions between pyridinone moiety and surrounding amino acid residues, indicating that pyridinone moiety, not only provided chelating ability but also functioned as a critical piece in binding with MAO-B.

### Cognitive and memory assays in vivo

**Figure 7.**
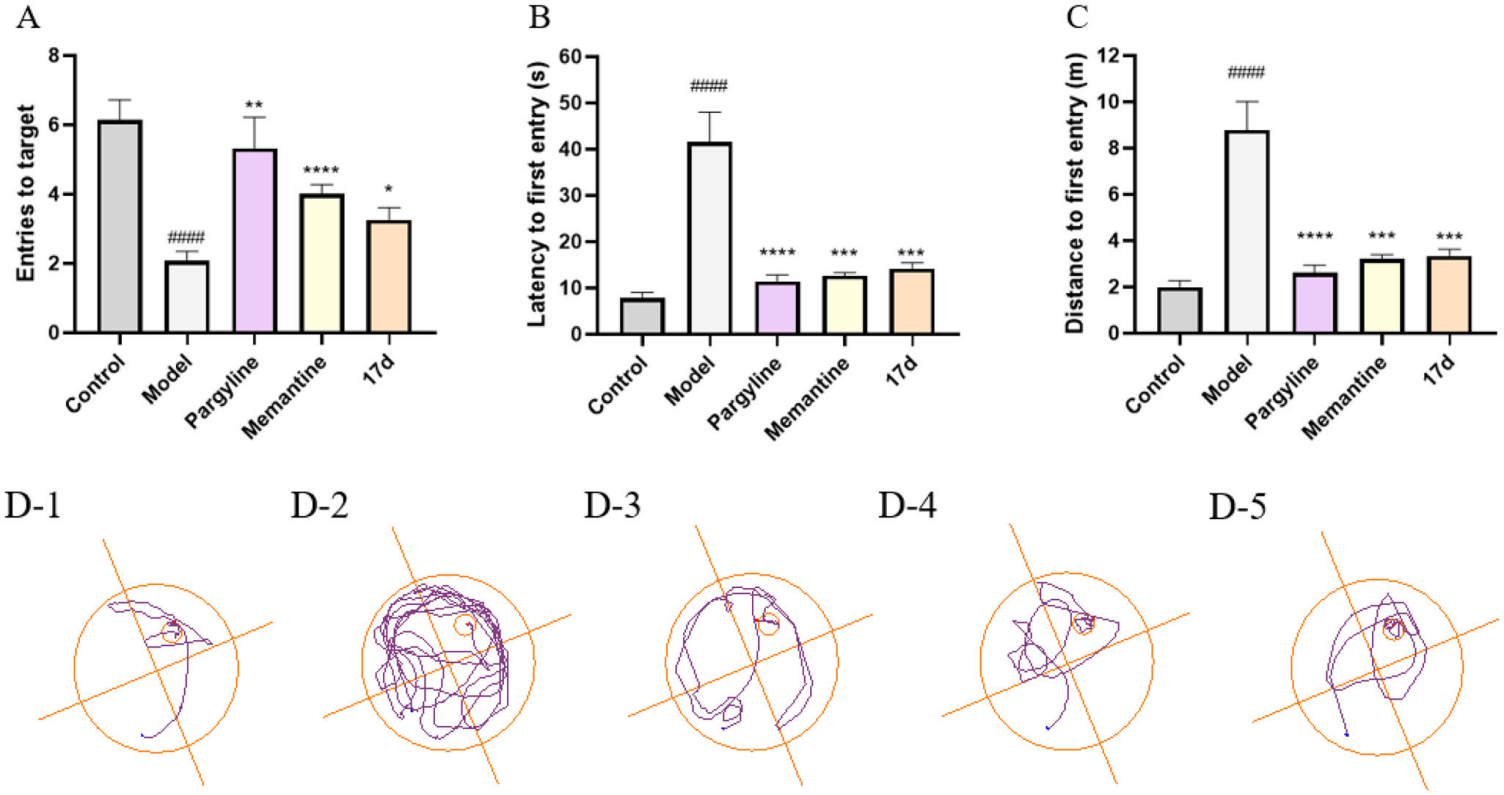
Pargyline (15 mg/kg), Memantine (15 mg/kg), and compound **17d** (15 mg/kg) were evaluated for scopolamine-induced (15 mg/kg) memory impairment in ICR mice in the Morris water maze. The mouse trajectories of the mice are shown as the control (**D–1**), model (**D–2**), pargyline (**D–3),** Memantine (**D–4)**, and **17d** (**D–5**) groups. Data are presented as the mean ± SEM (*n* = 15; ^#^*p* < 0.05, ^##^*p* < 0.01, ^###^*p* < 0.001, and ^####^*p* < 0.0001, Control group vs. scopolamine model group; *p < 0.05, **p < 0.01, ***p < 0.001, and ^****^p < 0.0001, Pargyline group, Memantine group, **17d** vs. scopolamine model group).

Towards this end, compound **17d** exhibited favourable multipotent activity. To further explore the *in vivo* anti-AD effect of compound **17d** on the scopolamine-induced cognitive impairment mice model, the Morris Water Maze experiment was selected; data are shown in Figure 7 and Table 5.

**Table 5.** Effects of compound **17d** (15 mg/kg) on scopolamine-induced memory impairment in ICR mice evaluated by the Morris water maze test.

Group	Entries to target	Latency to first entry	Distance to first entry
Control	6.1 ± 0.6	8 ± 1	2.0 ± 0.3
Model	2.1 ± 0.3^####^	42 ± 6^####^	8.9 ± 1.2^####^
**17d**	3.3 ± 0.3*	14 ± 1***	3.3 ± 0.3***
Pargyline	5.3 ± 0.9**	11 ± 1****	2.6 ± 0.3****
Memantine	4.0 ± 0.3****	13 ± 1***	3.2 ± 0 .2***

Pargyline (15 mg/kg) and Memantine (15 mg/kg) were used as reference.

Data are presented as the mean ± SEM (*n* = 15; ^####^*p* < 0.0001, Control group vs. scopolamine model group; *p < 0.05, **p < 0.01, ***p < 0.001, and ****p < 0.0001, Pargyline group, Memantine group, 17d vs. scopolamine model group).

According to the latency (Figure 7(B)) and distance (Figure 7(C)) to the first entry, the time (42 ± 6 vs. 8 ± 1, ^####^*p* < 0.0001) and distance (8.9 ± 1.2 vs. 2.0 ± 1.3, ^####^*p* < 0.0001) of reaching the platform of mice treated with scopolamine were significantly prolonged, indicating that the mice model of cognitive impairment has been well-established. According to the entries to target (Figure 7(A)), treatment with pargyline and memantine notably increased the entries they crossed the platform (5.3 ± 0.9 vs. 2.1 ± 0.3, ^#^*p* < 0.05; 4.0 ± 0.3 vs. 2.1 ± 0.3, ^####^*p* < 0.0001). As shown in Figure 7(B), treatment with pargyline markedly improved the cognitive impairment and reduced the latency to reach the platform compared with the model group (11 ± 1 vs. 42 ± 6, ^****^p < 0.0001), so as memantine (13 ± 1 vs. 42 ± 6, ***p < 0.001). Furthermore, the latency to target was significantly shortened when treated with **17d** (14 ± 1 vs. 42 ± 6, ***p < 0.001). In terms of the distance to the first entry (Figure 7(C)), mice of the pargyline group led to a remarkably shorter distance to the first entry (2.6 ± 0.3 vs. 8.9 ± 1.2, ^****^p < 0.0001). The group of **17d** exhibited a comparable activity to pargyline (3.3 ± 0.3 vs. 2.6 ± 0.3).

**Figure 8.**
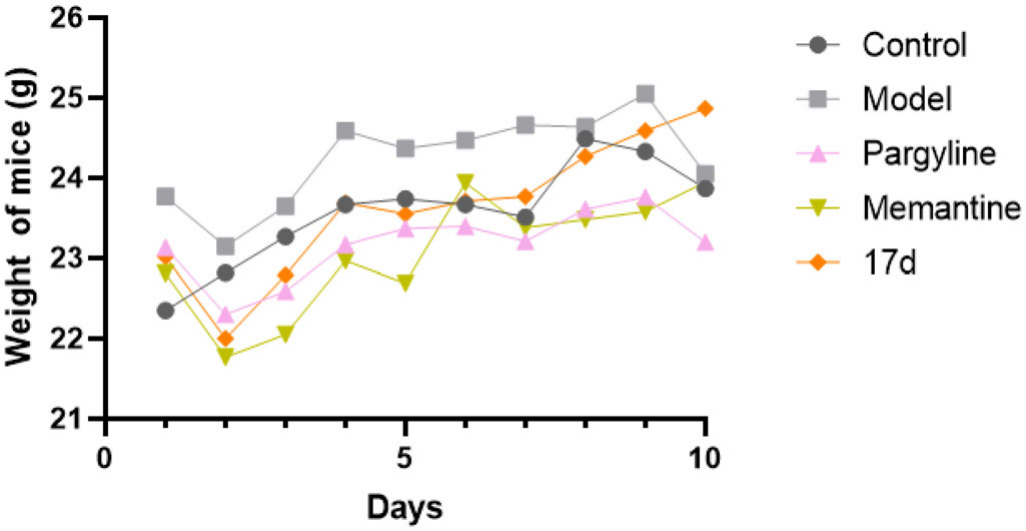
The pH-dependent UV spectra of compound **17d**. (A) The pH-dependence of the titration spectrum of compound **17d**. (B) The p*K*_a_ values of compound **17d** over the pH range of 1.3–11.0 in 0.1 M KCl at 25 °C.

According to the trajectory diagram (Figure 7(D)), the trajectory of pargyline, memantine, and compound **17d** were very clear. In contrast, the trajectory of the model group was very chaotic, which demonstrated that the scopolamine-induced cognitive impairment was significantly ameliorated by compound **17d.** As illustrated in Figure 8, except for the control group, all groups of mice showed a significant decrease in body weight after administration on the first day. However, the body weight of mice in group **17d** increased after ten days, partially indicating that **17d** had no obvious cytotoxicity.

### Pharmacokinetic property and BBB permeability investigation

UHPLC-MS/MS method was adopted to investigate the pharmacokinetic profiles of its metabolites after intravenous (i.v.) administration (2.34 mg/kg) and intragastric (i.g.) administration (15 mg/kg) of **17d** in the tail vein of rats. The main plasma pharmacokinetic parameters are listed in Table 6. As we can see, compound **17d** has higher bioavailability in the case of gavage administration (*F* = 9.25%), and can be absorbed quickly (*T*_max_ = 0.25 h) after i.g. administration. Although shorter half-time and lower brain districts, the above results indicated that **17d** would be instructive for further study.

**Table 6.** Pharmacokinetic parameters of **17d** after its administration^a^.

Administration	i.v. (2.34 mg/kg)	i.g. (15 mg/kg)
AUC_0–24_ (ng/ml*h)	745.23 ± 88.38	439.36 ± 59.60
AUC_0–∞_ (n /ml*h)	748.74 ± 87.10	443.82 ± 65.33
MRT_0–∞_ (h)	0.25 ± 0.08	0.81 ± 0.09
*t*_1/2_ (h)	0.32 ± 0.00	0.51 ± 0.18
CL (L/h/kg)	3.15 ± 0.37	0.03 ± 0.01
*V* (L/kg)	1.46 ± 0.19	0.02 ± 0.01
*C*_max_ (ng/ml)	2153.83 ± 636.73	389.51 ± 50.09
*T*_max_ (h)	0.083 ± 0	0.25 ± 0.00
F (%)	–	9.25%
Brain **17d** content (ng/g)^b^	–	6.02 ± 0.08

^a^Values are expressed as the mean ± SD (*n* = 3).

^b^Data collected from *C*_max_ time point.

## Conclusion

Herein, a novel series of chromone-pyridinone hybrids as dual-target-directed agents were designed and synthesised by pharmacophore fusion strategy. The designed derivatives displayed preferable biometal chelating effects and anti-MAO-B activities. Compound **17d** was proved to be the most potent MAO-B inhibitor (*h*MAO-B IC_50_ = 67.02 ± 4.3 nM), outperforming the reference drug pargyline (*h*MAO-B IC_50_ = 111.3 ± 0.6 nM). The molecular docking research revealed that **17d** may bind to both the substrate cavity and the entrance cavity of MAO-B. Furthermore, compound **17d** was demonstrated as a noncytotoxic agent and was able to considerably ameliorate cognitive dysfunction in a scopolamine-induced mice cognition-impaired model. Despite its undesired PK property, **17d** remains a promising multifaceted agent that is worth further investigation.

## Experimental section

### General information

Unless otherwise indicated, all reagents and solvents were used without further purification acquired from commercial suppliers. NMR spectra were acquired on Bruker and Varian spectrometers at 600, 400 MHz for ^1^H and 150, 100 MHz for ^13^C, respectively. Melting points (m.p., uncorrected) were measured with a Büchi B-540 m.p. apparatus. High-resolution mass spectra (HRMS) were recorded with a Shimadzu LCMS-IT-TOF mass spectrometer equipped with an electrospray ionisation (ESI) source. Routinely, the procedure of reactions was monitored on silica gel by thin-layer chromatography. The purity of the final compounds (>97%) was verified by high-performance liquid chromatography (HPLC) equipped with a UV-diode array detector.

### General procedure for the preparation of intermediate 4f–l

Anhydrous potassium carbonate (7.5 mmol) and compound **9** (5 mmol) were dissolved in acetone (20 ml), and appropriately substituted benzyl bromide derivatives (5.5 mmol) were added to the mixture and refluxed for 8 h. Upon completion, the solution of reaction was filtered and the filtrate was removed under reduced pressure. The target compounds were purified by recrystallisation with MeOH/DCM to yield solid powder **4f–l.**

#### 1–(4-(Benzyloxy)-2-hydroxyphenyl)ethan-1-one (4f)

Yield: 87%, white solid, m.p. = 105–107 °C; ^1^H NMR (400 MHz, CDCl_3_) *δ* 12.73 (s, 1H), 7.64 (d, *J* = 8.4 Hz, 1H), 7.46–7.34 (m, 5H), 6.53–6.50 (m, 2H), 5.10 (s, 2H), 2.56 (s, 3H); ^13^C NMR (100 MHz, CDCl_3_) *δ* 202.7, 165.3, 136.0, 132.5, 128.9, 128.5, 127.7, 114.2, 108.3, 102.0, 70.4, 26.4.

#### 1–(2-Hydroxy-4-((3-methylbenzyl)oxy)phenyl)ethan-1-one (4g)

Yield: 52%, white solid, m.p. = 98–99 °C; ^1^H NMR (400 MHz, CDCl_3_) *δ* 12.74 (s, 1H), 7.64 (d, *J* = 8.4 Hz, 1H), 7.29 (t, *J* = 7.6 Hz, 1H), 7.23 (d, *J* = 4.8 Hz, 2H), 7.16 (d, *J* = 7.6 Hz, 1H), 6.53–6.05 (m, 2H), 5.05 (s, 2H), 2.56 (s, 3H), 2.38 (s, 3H); ^13^C NMR (100 MHz, CDCl_3_) *δ* 202.7, 165.4, 165.3, 138.6, 135.9, 132.5, 129.2, 128.8, 128.4, 124.8, 114.2, 108.3, 102.0, 70.4, 26.3, 21.5.

#### 1–(2-Hydroxy-4-((4-methylbenzyl)oxy)phenyl)ethan-1-one (4h)

Yield: 83%, white solid, m.p. = 99–101 °C; ^1^H NMR (400 MHz, CDCl_3_) *δ* 12.73 (s, 1H), 7.63 (d, *J* = 9.6 Hz, 1H), 7.31 (d, *J* = 7.6 Hz, 2H), 7.20 (d, *J* = 7.6 Hz, 2H), 6.52–6.49 (m, 2H), 5.05 (s, 2H), 2.55 (s, 3H), 2.37 (s, 3H); ^13^C NMR (100 MHz, CDCl_3_) *δ* 202.7, 165.4, 165.3, 138.3, 133.0, 132.4, 129.5, 127.8, 114.2, 108.3, 102.0, 70.3, 26.3, 21.3.

#### 1–(4-((3-Fluorobenzyl)oxy)-2-hydroxyphenyl)ethan-1-one (4i)

Yield: 93%, white solid, m.p. = 108–110 °C; ^1^H NMR (400 MHz, CDCl_3_) *δ* 12.72 (s, 1H), 7.65 (d, *J* = 8.8 Hz, 1H), 7.36 (q, *J* = 7.2 Hz, 1H), 7.18 (d, *J* = 7.6 Hz, 1H), 7.13 (d, *J* = 9.2 Hz, 1H), 7.03 (t, *J* = 8.4 Hz, 1H), 6.51 (dd, *J* = 8.8, 2.0 Hz, 1H), 6.47 (d, *J* = 1.2 Hz, 1H), 5.09 (s, 2H), 2.56 (s, 3H); ^13^C NMR (100 MHz, CDCl_3_) *δ* 202.7, 165.3, 164.9, 163.1 (d, *^1^J* = 245 Hz), 138.6 (d, *^3^J* = 7 Hz), 132.5, 130.4 (d, *^3^J* = 8 Hz), 122.9 (d, *^4^J* = 3 Hz), 115.3 (d, *^2^J* = 21 Hz), 114.4 (d, *^2^J* = 22 Hz), 114.4, 108.2, 102.0, 69.4, 26.3.

#### 1–(4-((4-Fluorobenzyl)oxy)-2-hydroxyphenyl)ethan-1-one (4j)

Yield: 92%, white solid, m.p. = 115–117 °C; ^1^H NMR (400 MHz, CDCl_3_) *δ* 12.73 (s, 1H), 7.64 (d, *J* = 8.8 Hz, 1H), 7.39 (dd, *J* = 8.0, 5.6 Hz, 2H), 7.08 (t, *J* = 8.8 Hz, 2H), 6.52–6.48 (m, 2H), 5.04 (s, 2H), 2.55 (s, 3H); ^13^C NMR (100 MHz, CDCl_3_) *δ* 202.7, 165.3, 165.1, 162.8 (d, *^1^J* = 245 Hz), 132.5, 131.8 (d, *^4^J* = 3 Hz), 129.5 (d, *^3^J* = 8 Hz), 115.8 (d, *^2^J* = 22 Hz), 114.3, 108.2, 102.0, 69.6, 26.3.

#### 1–(4-((3-Chlorobenzyl)oxy)-2-hydroxyphenyl)ethan-1-one (4k)

Yield: 70%, white solid, m.p. = 127–129 °C; ^1^H NMR (400 MHz, CDCl_3_) *δ* 12.72 (s, 1H), 7.64 (d, *J* = 8.8 Hz, 1H), 7.41 (s, 1H), 7.35–7.26 (m, 3H), 6.51 (dd, *J* = 8.8, 2.0 Hz, 1H), 6.46 (d, *J* = 2.0 Hz, 1H), 5.06 (s, 2H), 2.55 (s, 3H); ^13^C NMR (100 MHz, CDCl_3_) *δ* 202.8, 165.3, 164.9, 138.1, 134.8, 132.6, 130.1, 128.6, 127.6, 125.5, 114.4, 108.1, 102.0, 69.4, 26.3.

#### 1–(4-((4-Chlorobenzyl)oxy)-2-hydroxyphenyl)ethan-1-one (4l)

Yield: 79%, white solid, m.p. = 102–104 °C; ^1^H NMR (400 MHz, CDCl_3_) *δ* 12.72 (s, 1H), 7.64 (d, *J* = 8.8 Hz, 1H), 7.37 (d, *J* = 9.2 Hz, 2H), 7.34 (d, *J* = 9.2 Hz, 2H), 6.50 (dd, *J* = 8.8, 2.0 Hz, 1H), 6.47 (d, *J* = 2.0 Hz, 1H), 5.05 (s, 2H), 2.56 (s, 3H); ^13^C NMR (100 MHz, CDCl_3_) *δ* 202.8, 165.3, 165.0, 134.5, 134.3, 132.5, 129.0, 129.0, 114.4, 108.2, 102.0, 69.5, 26.4.

### General procedure for the preparation of intermediates 5a–l and 6a–e

A solution of compounds **4a–l** (10 mmol) in anhydrous DMF (20 ml) was stirred at −10 °C for 35 min. Subsequently, phosphoryl chloride (20 mmol) was added dropwise for 20 min at −10 °C. Then the reaction mixture was allowed to stir at room temperature for another 15 h before being poured into ice water. The produced residue was filtered and washed with ethyl ether to obtain solid powders **5a–l**. Afterward, a mixture of compounds **5a–e** (3 mmol) in methylene chloride (40 ml) and sulphamic acid (15 mmol) in water (36 ml) was cooled to −0 °C. Sodium chloride (12 mmol) was dissolved in water (26 ml) and added dropwise to the mixture slowly at −0 °C. After 12 h, the reaction solution was extracted with DCM three times. The organic extracts were combined, dried with anhydrous Na_2_SO_4_, and evaporated. The products **6a–e** were finally purified by recrystallisation with methanol.

#### 4-Oxo-4H-chromene-3-carbaldehyde (5a)

Yield: 72%, yellow solid, m.p. = 149–151 °C; ^1^H NMR (400 MHz, CDCl_3_) *δ* 10.39 (s, 1H), 8.55 (s, 1H), 8.30 (dd, *J* = 8.0, 1.2 Hz, 1H), 7.78–7.73 (m, 1H), 7.55–7.48 (m, 2H); ^13^C NMR (100 MHz, CDCl_3_) *δ*188.7, 176.1, 160.8, 156.3, 135.0, 126.8, 126.3, 125.5, 120.5, 118.8.

#### 7-Methyl-4-oxo-4H-chromene-3-carbaldehyde (5b)

Yield: 73%, yellow solid, m.p. = 181–183 °C; ^1^H NMR (400 MHz, CDCl_3_) *δ* 10.31 (d, *J* = 4.4 Hz, 1H), 8.44 (d, *J* = 2.8 Hz, 1H), 8.12–8.08 (m, 1H), 7.26 (s, 1H), 7.23 (d, *J* = 6.0 Hz, 1H), 2.45 (s, 3H); ^13^C NMR (100 MHz, CDCl_3_) *δ* 188.8, 175.9, 160.5, 156.4, 146.6, 128.1, 126.0, 123.1, 120.4, 118.5, 22.0.

#### 6-Methyl-4-oxo-4H-chromene-3-carbaldehyde (5c)

Yield: 84%, yellow solid, m.p. = 171–173 °C; ^1^H NMR (400 MHz, CDCl_3_) *δ* 10.36 (s, 1H), 8.51 (s, 1H), 8.05 (s, 1H), 7.54 (dd, *J* = 8.8, 1.6 Hz, 1H), 7.41 (d, *J* = 8.4 Hz, 1H), 2.47 (s, 3H); ^13^C NMR (100 MHz, CDCl_3_) *δ* 188.9, 176.2, 160.6, 154.6, 137.0, 136.1, 125.6, 125.1, 120.3, 118.5, 21.1.

#### 7- Methoxy −4-oxo-4H-chromene-3-carbaldehyde (5d)

Yield: 65%, brown solid, m.p. = 148–150 °C; ^1^H NMR (400 MHz, CDCl_3_) *δ* 10.37 (s, 1H), 8.47 (s, 1H), 8.19 (d, *J* = 8.8 Hz, 1H), 7.04 (dd, *J* = 8.8, 2.0 Hz, 1H), 6.91 (d, *J* = 2.0 Hz, 1H), 3.92 (s, 3H); ^13^C NMR (100 MHz, CDCl_3_) *δ* 189.0, 175.4, 165.0, 160.3, 158.1, 127.7, 120.4, 119.0, 115.6, 101.3, 56.2.

#### 6-Methoxy − 4-oxo-4H-chromene-3-carbaldehyde (5e)

Yield: 91%, yellow solid, m.p. = 162–164 °C; ^1^H NMR (400 MHz, CDCl_3_) *δ* 10.38 (s, 1H), 8.51 (s, 1H), 7.62 (d, *J* = 2.8 Hz, 1H), 7.45 (d, *J* = 9.2 Hz, 1H), 7.30 (dd, *J* = 9.2, 3.2 Hz, 1H), 3.91 (s, 3H); ^13^C NMR (100 MHz, CDCl_3_) *δ* 188.8, 176.0, 160.3, 158.1, 151.1, 126.3, 124.6, 120.1, 119.7, 105.6, 56.2.

#### 7-(Benzyloxy)-4-oxo-4H-chromene-3-carbaldehyde (5f)

Yield: 82%, brown solid, m.p. = 126–128 °C; ^1^H NMR (400 MHz, CDCl_3_) *δ* 10.37 (s, 1H), 8.46 (s, 1H), 8.21 (d, *J* = 9.2 Hz, 1H), 7.43 (d, *J* = 6.0 Hz, 5H), 7.12 (dd, *J* = 9.2, 2.0 Hz, 1H), 6.98 (d, *J* = 1.6 Hz, 1H), 5.18 (s, 2H); ^13^C NMR (100 MHz, CDCl_3_) *δ* 189.0, 175.4, 164.0, 160.3, 158.0, 135.4, 129.0, 128.7, 127.7, 127.6, 120.4, 119.2, 116.2, 102.4, 70.9.

#### 7-((3-Methylbenzyl)oxy)-4-oxo-4H-chromene-3-carbaldehyde (5g)

Yield: 77%, brown solid, m.p. = 144–146 °C; ^1^H NMR (400 MHz, CDCl_3_) *δ* 10.37 (s, 1H), 8.47 (s, 1H), 8.20 (d, *J* = 8.9 Hz, 1H), 7.30 (td, *J* = 7.4, 2.5 Hz, 3H), 7.13 (d, *J* = 2.3 Hz, 1H), 7.11 (d, *J* = 2.3 Hz, 1H), 6.98 (d, *J* = 2.3 Hz, 1H), 5.14 (s, 2H), 2.37 (s, 3H); ^13^C NMR (100 MHz, CDCl_3_) *δ* 188.9, 175.3, 164.0, 160.3, 157.9, 138.7, 135.2, 129.4, 128.8, 128.3, 127.6, 124.7, 120.2, 119.0, 116.1, 102.2, 70.9, 21.5.

#### 7-((4-Methylbenzyl)oxy)-4-oxo-4H-chromene-3-carbaldehyde (5h)

Yield: 78%, brown solid, m.p. = 158–160 °C; ^1^H NMR (400 MHz, CDCl_3_) *δ*10.37 (s, 1H), 8.47 (s, 1H), 8.19 (d, *J* = 8.9 Hz, 1H), 7.33 (d, *J* = 7.9 Hz, 2H), 7.22 (d, *J* = 7.9 Hz, 2H), 7.11 (dd, *J* = 8.9, 2.3 Hz, 1H), 6.98 (d, *J* = 2.2 Hz, 1H), 5.13 (s, 2H), 2.37 (s, 3H); ^13^C NMR (150 MHz, CDCl_3_) *δ* 188.8, 175.2, 163.9, 160.1, 157.8, 138.5, 132.2, 129.5, 127.6, 127.5, 120.2, 118.9, 116.0, 102.1, 70.7, 21.2.

#### 7-((3-Fluorobenzyl)oxy)-4-oxo-4H-chromene-3-carbaldehyde (5i)

Yield: 95%, brown solid, m.p. = 119–121 °C; ^1^H NMR (400 MHz, CDCl_3_) *δ* 10.36 (s, 1H), 8.46 (s, 1H), 8.21 (d, *J* = 8.8 Hz, 1H), 7.38 (d, *J* = 6.0 Hz, 1H), 7.21 (d, *J* = 7.2 Hz, 1H), 7.15 (d, *J* = 9.2 Hz, 1H), 7.11 (d, J = 8.8 Hz, 1H), 7.06 (s, 1H), 6.97 (s, 1H), 5.17 (s, 2H); ^13^C NMR (100 MHz, CDCl_3_) *δ* 188.9, 175.3, 163.1 (d, *^1^J* = 246 Hz), 163.7, 160.4, 158.0, 130.6 (d, *^3^J* = 8 Hz), 127.8, 122.9 (d, *^4^J* = 2 Hz), 120.4, 119.4, 116.0, 115.6 (d, *^2^J* = 20 Hz), 115.4 (d, *^4^J* = 2 Hz), 114.4 (d, *^2^J* = 22 Hz), 102.4, 70.0.

#### 7-((4-Fluorobenzyl)oxy)-4-oxo-4H-chromene-3-carbaldehyde (5j)

Yield: 75%, brown solid, m.p. = 118–120 °C; ^1^H NMR (400 MHz, CDCl_3_) *δ* 10.36 (s, 1H), 8.46 (s, 1H), 8.20 (d, *J* = 8.8 Hz, 1H), 7.43 (d, *J* = 5.6 Hz, 1H), 7.41 (d, *J* = 5.6 Hz, 1H), 7.11 (dd, *J* = 5.2, 2.8 Hz, 2H), 7.09 (d, *J* = 2.8 Hz, 1H), 6.97 (d, *J* = 2.0 Hz, 1H), 5.13 (s, 2H); ^13^C NMR (100 MHz, CDCl_3_) *δ* 188.9, 175.3, 162.9 (d, *^1^J* = 246 Hz), 163.8, 160.4, 158.0, 131.2 (d, *^4^J* = 3 Hz), 129.6 (d, *^3^J* = 9 Hz), 127.8, 120.4, 119.3, 116.1, 115.8, 102.4, 70.2.

#### 7-((3-Chlorobenzyl)oxy)-4-oxo-4H-chromene-3-carbaldehyde (5k)

Yield: 94%, brown solid, m.p. = 110–112 °C; ^1^H NMR (400 MHz, CDCl_3_) *δ* 10.37 (s, 1H), 8.47 (s, 1H), 8.22 (d, *J* = 8.8 Hz, 1H), 7.44 (s, 1H), 7.36–7.33 (m, 3H), 7.12 (dd, *J* = 8.8, 2.4 Hz, 1H), 6.97 (d, *J* = 2.4 Hz, 1H), 5.15 (s, 2H); ^13^C NMR (150 MHz, CDCl_3_) *δ* 188.7, 175.2, 163.5, 160.2, 157.8, 137.2, 134.8, 130.1, 128.7, 127.7, 127.4, 125.3, 120.2, 119.2, 115.8, 102.2, 69.8.

#### 7*-((4-Chlorobenzyl)oxy)-4-oxo-4H-chromene-3-carbaldehyde (5l)*

Yield: 80%, brown solid, m.p. = 111–113 °C; ^1^H NMR (400 MHz, CDCl_3_) *δ* 10.35 (s, 1H), 8.46 (s, 1H), 8.20 (d, *J* = 8.8 Hz, 1H), 7.38 (s, 4H), 7.10 (dd, *J* = 8.8, 2.4 Hz, 1H), 6.96 (d, *J* = 2.0 Hz, 1H), 5.13 (s, 2H); ^13^C NMR (100 MHz, CDCl_3_) *δ* 188.9, 175.3, 163.7, 160.4, 157.9, 133.8, 132.5, 129.1, 129.0, 127.8, 120.3, 119.2, 116.0, 102.3, 70.1.

#### 4-Oxo-4H-chromene-3-carboxylic acid (6a)

Yield: 89%, yellow solid, m.p. = 201–202 °C; ^1^H NMR (400 MHz, DMSO-d_6_) *δ* 13.21 (s, 1H), 9.10 (s, 1H), 8.15 (dd, *J* = 8.0, 1.2 Hz, 1H), 7.91 (t, *J* = 8.4 Hz, 1H), 7.77 (d, *J* = 8.4 Hz, 1H), 7.60 (t, *J* = 7.6 Hz, 1H); ^13^C NMR (100 MHz, DMSO-d_6_) *δ* 176.1, 163.8, 163.7, 155.7, 135.5, 126.8, 125.4, 123.3, 118.8, 114.3.

#### 7-Methyl-4-oxo-4H-chromene-3-carboxylic acid (6b)

Yield: 76%, yellow solid, m.p. = 206–208 °C; ^1^H NMR (400 MHz, DMSO-d_6_) *δ* 13.29 (s, 1H), 9.08 (s, 1H), 8.03 (d, *J* = 7.2 Hz, 1H), 7.60 (s, 1H), 7.43 (d, *J* = 6.8 Hz, 1H), 2.50 (s, 3H); ^13^C NMR (100 MHz, DMSO-d_6_) *δ* 176.3, 163.8, 163.7, 155.9, 147.1, 128.2, 125.1, 120.8, 118.3, 113.8, 21.3.

#### 6-Methyl-4-oxo-4H-chromene-3-carboxylic acid (6c)

Yield: 85%, yellow solid, m.p. = 248–250 °C; ^1^H NMR (400 MHz, DMSO-d_6_) *δ* 13.28 (s, 1H), 9.11 (s, 1H), 7.96 (s, 1H), 7.75 (dd, J = 8.4, 1.2 Hz,1H), 7.70 (d, J = 8.8 Hz, 1H), 2.46 (s, 3H); ^13^C NMR (100 MHz, DMSO-d_6_) *δ* 176.3, 163.9, 163.7, 154.1, 136.8, 136.7, 124.6, 123.0, 118.6, 114.0, 20.5.

#### 7-Methoxy-4-oxo-4H-chromene-3-carboxylic acid (6d)

Yield: 48%, yellow solid, m.p. = 176–178 °C; ^1^H NMR (400 MHz, DMSO-d_6_) *δ*13.24 (s, 1H), 9.08 (s, 1H), 8.06 (d, *J* = 8.8 Hz, 1H), 7.29 (s, 1H), 7.18 (d, *J* = 8.0 Hz, 1H), 3.93 (s, 3H); ^13^C NMR (100 MHz, DMSO-d_6_) *δ* 176.2, 165.0, 163.8, 163.7, 157.9, 126.8, 116.5, 116.2, 113.4, 101.3, 56.4.

#### 6-Methoxy-4-oxo-4H-chromene-3-carboxylic acid (6e)

Yield: 78%, yellow solid, m.p. = 172–174 °C; ^1^H NMR (400 MHz, DMSO-d_6_) *δ* 13.30 (s, 1H), 9.12 (s, 1H), 7.78 (d, *J* = 8.8 Hz, 1H), 7.54 (d, *J* = 2.8 Hz, 1H), 7.51 (s, 1H), 3.92 (s, 3H); ^13^C NMR (100 MHz, DMSO-d_6_) *δ* 176.1, 163.9, 163.4, 157.4, 150.5, 124.6, 124.0, 120.4, 113.2, 105.1, 55.9.

### General procedure for the preparation of compounds 7a–e

The intermediates **6a–e** (1 mmol) were dissolved in DMF (4 ml), then POCl_3_ (1.3 mmol) was added to the mixture, and stirred for 40 min at room temperature for the generation of corresponding acyl chloride. Subsequently, intermediate **3** (1 mmol) was added. After 10 h, the reaction mixture was diluted with CH_2_Cl_2_, washed with water two times, and with saturated NaHCO_3_ solution three times. The combined organic phases were dried over Na_2_SO_4_ and concentrated *in vacuo*. The target product was purified by column chromatography DCM/MeOH, and the solvents were removed under reduced pressure to give **7a–e**.

#### N-(2–(3-((4-methoxybenzyl)oxy)-2-methyl-4-oxopyridin-1(4H)-yl)ethyl)-4-oxo-4H-chromene-3-carboxamide (7a)

Yield: 57%, yellow oil, ^1^H NMR (400 MHz, CDCl_3_) *δ* 9.52 (t, *J* = 5.2 Hz, 1H), 8.93 (s, 1H), 8.26 (d, *J* = 8.0 Hz, 1H), 7.78 (t, *J* = 8.4 Hz, 1H), 7.57 (d, *J* = 8.4 Hz, 1H), 7.52 (t, *J* = 7.6 Hz, 1H), 7.32 (d, *J* = 8.4 Hz, 2H), 7.19 (d, *J* = 7.6 Hz, 1H), 6.84 (d, *J* = 8.4 Hz, 2H), 6.38 (d, *J* = 7.2 Hz, 1H), 5.15 (s, 2H), 4.00 (t, *J* = 6.4 Hz, 2H), 3.78 (s, 3H), 3.61 (q, *J* = 6.4 Hz, 2H), 2.14 (s, 3H); ^13^C NMR (100 MHz, CDCl_3_) *δ* 177.2, 173.7, 163.9, 162.6, 159.6, 156.3, 146.3, 140.9, 138.5, 135.1, 131.0, 130.0, 126.7, 126.4, 124.2, 118.6, 117.5, 115.3, 113.7, 72.8, 55.4, 52.3, 39.6, 12.6; ESI-HRMS: *m/z* calcd for C_26_H_24_N_2_O_6_ [M + H]^+^: 461.1707; found: 461.1674.

#### N-(2–(3-((4-methoxybenzyl)oxy)-2-methyl-4-oxopyridin-1(4H)-yl)ethyl)-7-methyl-4-oxo-4H-chromene-3-carboxamide (7b)

Yield: 36%, yellow oil, ^1^H NMR (400 MHz, CDCl_3_) *δ* 9.57 (t, *J* = 5.8 Hz, 1H), 8.89 (s, 1H), 8.13 (d, *J* = 8.2 Hz, 1H), 7.37 − 7.30 (m, 4H), 7.22 (d, *J* = 7.5 Hz, 1H), 6.87 − 6.83 (m, 2H), 6.42 (d, *J* = 7.5 Hz, 1H), 5.15 (s, 2H), 4.01 (t, *J* = 6.7 Hz, 2H), 3.78 (s, 3H), 3.61 (q, *J* = 6.5 Hz, 2H), 2.53 (s, 3H), 2.15 (s, 3H); ^13^C NMR (100 MHz, CDCl_3_) *δ* 177.0, 173.6, 164.0, 162.3, 159.5, 156.3, 146.8, 146.1, 141.0, 138.5, 130.8, 129.8, 128.1, 126.0, 121.8, 118.2, 117.4, 114.9, 113.6, 72.7, 55.2, 52.2, 39.4, 21.9, 12.5.

#### N-(2–(3-((4-methoxybenzyl)oxy)-2-methyl-4-oxopyridin-1(4H)-yl)ethyl)-6-methyl-4-oxo-4H-chromene-3-carboxamide (7c)

Yield: 38%, yellow oil, ^1^H NMR (400 MHz, CDCl_3_) *δ* 9.55 (t, *J* = 5.8 Hz, 1H), 8.89 (s, 1H), 8.01 (s, 1H), 7.57 (dd, *J* = 8.6, 2.1 Hz, 1H), 7.45 (d, *J* = 8.6 Hz, 1H), 7.31 (d, *J* = 8.6 Hz, 2H), 7.20 (d, *J* = 7.5 Hz, 1H), 6.83 (d, *J* = 8.6 Hz, 2H), 6.37 (d, *J* = 7.5 Hz, 1H), 5.12 (s, 2H), 3.99 (t, *J* = 6.6 Hz, 2H), 3.77 (s, 3H), 3.60 (q, *J* = 6.4 Hz, 2H), 2.48 (s, 3H), 2.13 (s, 3H); ^13^C NMR (100 MHz, CDCl_3_) *δ* 177.1, 173.6, 164.0, 162.3, 159.5, 154.5, 146.1, 140.9, 138.5, 136.9, 136.2, 130.8, 129.8, 125.5, 123.7, 118.2, 117.4, 114.9, 113.6, 72.7, 55.2, 52.2, 39.4, 21.1, 12.5.

#### 7-Methoxy-N-(2–(3-((4-methoxybenzyl)oxy)-2-methyl-4-oxopyridin-1(4H)-yl) ethyl)-4-oxo-4H-chromene-3-carboxamide (7d)

Yield: 32%, yellow oil, ^1^H NMR (400 MHz, CDCl_3_) *δ* 9.62 (t, *J* = 5.8 Hz, 1H), 8.85 (s, 1H), 8.15 (d, *J* = 9.0 Hz, 1H), 7.33 (d, *J* = 8.6 Hz, 2H), 7.21 (d, *J* = 7.5 Hz, 1H), 7.07 (dd, *J* = 9.0, 2.4 Hz, 1H), 6.93 (d, *J* = 2.3 Hz, 1H), 6.88 − 6.83 (m, 2H), 6.41 (d, *J* = 7.5 Hz, 1H), 5.15 (s, 2H), 4.00 (t, *J* = 6.7 Hz, 2H), 3.94 (s, 3H), 3.79 (s, 3H), 3.61 (q, *J* = 6.5 Hz, 2H), 2.15 (s, 3H); ^13^C NMR (100 MHz, CDCl_3_) δ 176.4, 173.4, 165.1, 164.0, 162.0, 159.5, 158.0, 141.2, 138.6, 130.9, 130.9, 129.2, 128.0, 117.3, 117.3, 116.0, 114.9, 113.6, 100.5, 73.2, 56.1, 55.3, 52.2, 39.4, 12.5; ESI-HRMS: *m/z* calcd for C_27_H_26_N_2_O_7_ [M + H]^+^: 491.1813; found: 491.1826.

#### 6-Methoxy-N-(2–(3-((4-methoxybenzyl)oxy)-2-methyl-4-oxopyridin-1(4H)-yl) ethyl)-4-oxo-4H-chromene-3-carboxamide (7e)

Yield: 30%, yellow oil, ^1^H NMR (400 MHz, CDCl_3_) *δ* 9.57 (t, *J* = 5.7 Hz, 1H), 8.92 (s, 1H), 7.59 (d, *J* = 3.0 Hz, 1H), 7.51 (d, *J* = 9.2 Hz, 1H), 7.37 − 7.31 (m, 3H), 7.21 (d, *J* = 7.5 Hz, 1H), 6.87 − 6.82 (m, 2H), 6.41 (d, *J* = 7.5 Hz, 1H), 5.15 (s, 2H), 4.01 (t, *J* = 6.5 Hz, 2H), 3.92 (s, 3H), 3.79 (s, 3H), 3.62 (q, *J* = 6.3 Hz, 2H), 2.15 (s, 3H); ^13^C NMR (100 MHz, CDCl_3_) *δ* 176.8, 173.6, 164.0, 162.1, 159.5, 157.9, 151.0, 146.1, 141.0, 138.5, 130.9, 129.8, 124.9, 124.8, 119.9, 117.4, 114.3, 113.6, 105.3, 72.7, 56.0, 55.3, 52.2, 39.4, 12.6; ESI-HRMS: *m/z* calcd for C_27_H_26_N_2_O_7_ [M + H]^+^: 491.1813; found: 491.1821.

### General procedure for the preparation of compounds 8a–e, 15a–g, and 17a–l

The chromone derivatives **7a–e**, **14a–g**, and **16a–l** (0.3 mmol) were dissolved in anhydrous DCM (10 ml) and protected by nitrogen. After the solution was cooled to −48 °C, BCl_3_ (1 M in DCM, 1.5 eq) dissolved in anhydrous DCM (10 ml) was added dropwise slowly. After the addition was completed, the mixture was stirred for 12 h at room temperature. The excess boron trichloride was quenched with methanol (10 ml) and left to stir for another 1 h. After the removal of solvents in a high vacuum, the residues were purified by recrystallisation from methanol/ether to afford the final compounds **8a–e**, **15a–g**, and **17a–l** as solids.

#### N-(2–(3-hydroxy-2-methyl-4-oxopyridin-1(4H)-yl)ethyl)-4-oxo-4H-chromene-3-carboxamide (8a)

Yield: 93%, yellow solid, m.p. = 268–270 °C; ^1^H NMR (400 MHz, DMSO-d_6_) *δ* 9.30 (t, *J* = 6.0 Hz, 1H), 8.90 (s, 1H), 8.16 (d, *J* = 8.0 Hz, 1H), 8.09 (d, *J* = 6.8 Hz, 1H), 7.91 (t, *J* = 7.6 Hz, 1H), 7.76 (d, *J* = 8.4 Hz, 1H), 7.60 (t, *J* = 7.6 Hz, 1H), 7.30 (d, *J* = 6.8 Hz, 1H), 4.5 3 (t, *J* = 5.6 Hz, 2H), 4.01 (q, *J* = 6.0 Hz, 2H), 2.59 (s, 3H); ^13^C NMR (100 MHz, DMSO-d_6_) *δ* 176.0, 162.9, 162.7, 158.8, 155.6, 142.8, 141.8, 138.6, 135.3, 126.6, 125.4, 123.5, 118.69, 115.1, 110.5, 55.2, 38.3, 12.6; ESI-HRMS: *m/z* calcd for C_18_H_16_N_2_O_5_ [M + H]^+^: 341.1132; found: 341.1125. HPLC purity: 97.9%.

#### N-(2–(3-hydroxy-2-methyl-4-oxopyridin-1(4H)-yl)ethyl)-7-methyl-4-oxo-4H-chromene-3-carboxamide (8b)

Yield: 94.5%, yellow solid, m.p. = 270–272 °C; ^1^H NMR (400 MHz, DMSO-d_6_) *δ* 9.33 (t, *J* = 6.1 Hz, 1H), 8.95 (s, 1H), 8.07 (dd, *J* = 10.1, 7.6 Hz, 2H), 7.60 (s, 1H), 7.44 (d, *J* = 8.2 Hz, 1H), 7.24 (d, *J* = 6.8 Hz, 1H), 4.53 (t, *J* = 5.8 Hz, 2H), 3.78 (q, *J* = 5.9 Hz, 2H), 2.59 (s, 3H), 2.50 (s, 3H); ^13^C NMR (100 MHz, DMSO-d_6_) *δ* 175.8, 162.8, 162.7, 158.8, 155.7, 146.5, 142.8, 141.7, 138.5, 128.0, 125.2, 121.3, 118.2, 114.9, 110.5, 55.2, 38.2, 21.3, 12.6; ESI-HRMS: *m/z* calcd for C_19_H_18_N_2_O_5_ [M + H]^+^: 355.1288; found: 355.1276. HPLC purity: 98.9%.

#### N-(2–(3-hydroxy-2-methyl-4-oxopyridin-1(4H)-yl)ethyl)-6-methyl-4-oxo-4H-chromene-3-carboxamide (8c)

Yield: 98.5%, yellow solid, m.p. = 265–267 °C; ^1^H NMR (400 MHz, DMSO-d_6_) *δ* 9.32 (t, *J* = 6.1 Hz, 1H), 8.97 (s, 1H), 8.09 (d, *J* = 7.0 Hz, 1H), 7.95 (s, 1H), 7.74 (dd, *J* = 8.6, 1.9 Hz, 1H), 7.68 (d, *J* = 8.6 Hz, 1H), 7.29 − 7.23 (m, 1H), 4.54 (t, *J* = 5.8 Hz, 2H), 3.78 (q, *J* = 5.5, 5.1 Hz, 2H), 2.60 (s, 3H), 2.47 (s, 3H); ^13^C NMR (100 MHz, DMSO-d_6_) *δ* 176.4, 163.3, 163.2, 159.3, 154.4, 143.3, 142.3, 139.0, 136.9, 136.8, 125.0, 123.7, 119.0, 115.4, 111.0, 55.6, 38.7, 21.0, 13.1. ESI-HRMS: *m/z* calcd for C_19_H_18_N_2_O_5_ [M + H]^+^: 355.1288; found: 355.1273. HPLC purity: 99.1%.

#### N-(2–(3-hydroxy-2-methyl-4-oxopyridin-1(4H)-yl)ethyl)-7-methoxy-4-oxo-4H-chromene-3-carboxamide (8d)

Yield: 94.6%, yellow solid, m.p. = 248–250 °C; ^1^H NMR (400 MHz, DMSO-d_6_) *δ* 9.36 (t, *J* = 6.1 Hz, 1H), 8.91 (s, 1H), 8.06 (dd, *J* = 7.9, 3.3 Hz, 2H), 7.27 (d, *J* = 2.3 Hz, 1H), 7.21 (d, *J* = 6.9 Hz, 1H), 7.17 (dd, *J* = 8.9, 2.3 Hz, 1H), 4.52 (t, *J* = 5.7 Hz, 2H), 3.92 (s, 3H), 3.76 (q, *J* = 6.8, 6.3 Hz, 2H), 2.58 (s, 3H); ^13^C NMR (100 MHz, DMSO-d_6_) *δ* 175.7, 165.0, 163.3, 162.9, 159.3, 158.0, 143.3, 142.2, 139.0, 127.3, 117.6, 116.4, 115.3, 111.0, 101.5, 56.9, 55.6, 38.7, 13.1; ESI-HRMS: *m/z* calcd for C_19_H_18_N_2_O_6_ [M + H]^+^: 371.1238; found: 371.1227. HPLC purity: 99.7%.

#### N-(2–(3-hydroxy-2-methyl-4-oxopyridin-1(4H)-yl)ethyl)-6-methoxy-4-oxo-4H-chromene-3-carboxamide (8e)

Yield: 98.3%, yellow solid, m.p. = 260–262 °C; ^1^H NMR (400 MHz, DMSO-d_6_) *δ* 9.32 (t, *J* = 6.1 Hz, 1H), 8.97 (s, 1H), 8.08 (d, *J* = 7.0 Hz, 1H), 7.77 − 7.73 (m, 1H), 7.51 (dd, *J* = 7.3, 2.9 Hz, 2H), 7.24 (d, *J* = 7.0 Hz, 1H), 4.53 (t, *J* = 5.8 Hz, 2H), 3.89 (s, 3H), 3.78 (q, *J* = 6.0 Hz, 2H), 2.59 (s, 3H); ^13^C NMR (100 MHz, DMSO-d_6_) *δ* 176.1, 163.4, 163.0, 159.2, 157.8, 150.8, 143.3, 142.3, 139.0, 124.8, 124.6, 120.8, 114.8, 111.0, 105.8, 56.4, 55.7, 38.7, 13.1; ESI-HRMS: *m/z* calcd for C_19_H_18_N_2_NaO_6_ [M + Na]^+^: 393.1057; found: 393.1057. HPLC purity: 98.4%.

#### 7-(Benzyloxy)-N-(2–(3-hydroxy-2-methyl-4-oxopyridin-1(4H)-yl)ethyl)-4-oxo-4H-chromene-3-carboxamide (15a)

Yield: 54.7%, yellow solid, m.p. = 225–227 °C; ^1^H NMR (400 MHz, DMSO-d_6_) *δ* 9.37 (t, *J* = 6.1 Hz, 1H), 8.92 (s, 1H), 8.07 (t, *J* = 7.8 Hz, 2H), 7.50 (d, *J* = 7.0 Hz, 2H), 7.46 − 7.36 (m, 4H), 7.26 (dd, *J* = 8.9, 2.3 Hz, 1H), 7.17 (d, *J* = 6.9 Hz, 1H), 5.31 (s, 2H), 4.52 (t, *J* = 5.7 Hz, 2H), 3.77 (q, *J* = 7.4, 6.5 Hz, 2H), 2.59 (s, 3H); ^13^C NMR (100 MHz, DMSO-d_6_) *δ* 175.7, 164.0, 163.3, 163.0, 159.1, 157.9, 143.3, 142.3, 139.0, 136.3, 129.1, 128.7, 128.5, 127.4, 117.7, 116.9, 115.3, 110.9, 102.4, 70.8, 55.7, 38.7, 13.1; ESI-HRMS: *m/z* calcd for C_25_H_22_N_2_O_6_ [M + H]^+^: 447.1551; found: 447.1539. HPLC purity: 99.7%.

#### N-(2–(3-hydroxy-2-methyl-4-oxopyridin-1(4H)-yl)ethyl)-7-((3-methylbenzyl) oxy)-4-oxo-4H-chromene-3-carboxamide (15b)

Yield: 28%, yellow solid, m.p. = 238–240 °C; ^1^H NMR (400 MHz, DMSO-d_6_) *δ* 1H NMR (400 MHz, DMSO-d_6_) δ 9.37 (t, *J* = 6.1 Hz, 1H), 8.92 (s, 1H), 8.07 (dd, *J* = 7.9, 4.0 Hz, 2H), 7.36 − 7.34 (m, 1H), 7.33 − 7.21 (m, 5H), 7.17 (d, *J* = 6.9 Hz, 1H), 5.24 (s, 2H), 4.52 (t, *J* = 5.6 Hz, 2H), 3.76 (q, *J* = 5.6 Hz, 2H), 2.58 (s, 3H), 2.32 (s, 3H). ^13^C NMR (100 MHz, DMSO-d_6_) *δ* 175.3, 163.6, 162.9, 162.6, 158.9, 157.5, 142.9, 141.7, 138.6, 137.9, 135.8, 129.0, 128.6, 128.6, 127.0, 125.2, 117.3, 116.5, 114.9, 110.5, 102.0, 70.4, 55.2, 38.3, 21.1, 12.7. ESI-HRMS: *m/z* calcd for C_26_H_24_N_2_O_6_ [M + H]^+^: 461.1707; found: 461.1708. HPLC purity: 98.3%.

#### N-(2–(3-hydroxy-2-methyl-4-oxopyridin-1(4H)-yl)ethyl)-7-((4-methylbenzyl)oxy)-4-oxo-4H-chromene-3-carboxamide (15c)

Yield: 23%, yellow solid, m.p. = 234–236 °C; ^1^H NMR (400 MHz, DMSO-d_6_) *δ* 9.37 (t, *J* = 6.1 Hz, 1H), 8.92 (s, 1H), 8.05 (dd, *J* = 7.8, 5.3 Hz, 2H), 7.40 − 7.33 (m, 3H), 7.22 (d, *J* = 8.0 Hz, 3H), 7.15 (d, *J* = 6.9 Hz, 1H), 5.24 (s, 2H), 4.50 (t, *J* = 5.5 Hz, 2H), 3.77 − 3.73 (m, 2H), 2.57 (s, 3H), 2.31 (s, 3H). ^13^C NMR (100 MHz, DMSO-d_6_) *δ* 175.3, 163.6, 162.9, 162.6, 159.1, 157.5, 143.0, 141.4, 138.6, 137.7, 132.9, 129.2, 128.2, 127.0, 117.2, 116.5, 114.9, 110.5, 102.0, 70.3, 55.2, 38.3, 20.9, 12.6. ESI-HRMS: *m/*z calcd for C_26_H_24_N_2_O_6_ [M + H]^+^: 461.1707; found: 461.1724. HPLC purity: 98.2%.

#### 7-((3-Fluorobenzyl)oxy)-N-(2–(3-hydroxy-2-methyl-4-oxopyridin-1(4H)-yl)ethyl)-4-oxo-4H-chromene-3-carboxamide (15d)

Yield: 38%, yellow solid, m.p. = 237–239 °C; ^1^H NMR (400 MHz, DMSO-d_6_) *δ* 9.37 (t, *J* = 6.1 Hz, 1H), 8.93 (s, 1H), 8.08 (d, *J* = 8.7 Hz, 2H), 7.51 − 7.44 (m, 1H), 7.36 (dd, *J* = 11.7, 4.9 Hz, 3H), 7.29 − 7.18 (m, 3H), 5.33 (s, 2H), 4.53 (t, *J* = 5.7 Hz, 2H), 3.77 (q, *J* = 6.0 Hz, 2H), 2.59 (s, 3H). ^13^C NMR (100 MHz, DMSO-d_6_) *δ* 175.3, 163.3, 162.9, 162.6, 162.2 (d, ^1^*J* = 242 Hz), 158.7, 157.4, 142.9, 141.9, 138.8 (d, ^3^*J* = 8 Hz), 138.6, 130.7 (d, ^3^*J* = 8 Hz), 127.0, 123.9 (d, ^4^*J* = 2 Hz), 117.4, 116.4, 115.1 (d, ^2^*J* = 21 Hz), 114.9, 114.6 (d, ^2^*J* = 22 Hz), 110.5, 102.1, 69.4, 55.3, 38.3, 12.7. ESI-HRMS: *m/z* calcd for C_25_H_21_FN_2_O_6_ [M + H]^+^: 465.1456; found: 465.1475. HPLC purity: 98%.

#### 7-((4-Fluorobenzyl)oxy)-N-(2–(3-hydroxy-2-methyl-4-oxopyridin-1(4H)-yl) ethyl)-4-oxo-4H-chromene-3-carboxamide (15e)

Yield: 35%, yellow solid, m.p. = 246–248 °C; ^1^H NMR (400 MHz, DMSO-d_6_) *δ* 9.36 (t, *J* = 6.2 Hz, 1H), 8.91 (s, 1H), 8.07 (dd, *J* = 8.0, 3.9 Hz, 2H), 7.57 − 7.54 (m, 2H), 7.36 (d, *J* = 2.3 Hz, 1H), 7.27 − 7.23 (m, 3H), 7.19 (d, *J* = 6.9 Hz, 1H), 5.28 (s, 2H), 4.52 (t, *J* = 5.9 Hz, 2H), 3.76 (q, *J* = 6.0 Hz, 2H), 2.58 (s, 3H); ^13^C NMR (100 MHz, DMSO-d_6_) *δ* 175.7, 163.9, 163.7, 163.3, 163.0, 161.2, 159.1, 157.9, 143.3, 142.4, 139.1, 132.6 (d, ^4^*J* = 3 Hz), 130.9 (d, ^3^*J* = 9 Hz), 127.4, 116.9, 116.5 (d, ^1^*J* = 244 Hz), 115.9 (d, ^2^*J* = 22 Hz), 110.9, 102.4, 70.0, 55.7, 38.7, 13.1. ESI-HRMS: *m*/*z* calcd for C_25_H_22_FN_2_O_6_ [M + H]^+^: 465.1456; found: 465.1470. HPLC purity: 99.7%.

#### 7-((3-Chlorobenzyl)oxy)-N-(2–(3-hydroxy-2-methyl-4-oxopyridin-1(4H)-yl)ethyl)-4-oxo-4H-chromene-3-carboxamide (15f)

Yield: 55%, yellow solid, m.p. = 230–232 °C; ^1^H NMR (400 MHz, DMSO-d_6_) *δ* 9.36 (t, *J* = 6.1 Hz, 1H), 8.91 (s, 1H), 8.07 (d, *J* = 8.7 Hz, 2H), 7.57 (s, 1H), 7.45 (t, *J* = 4.4 Hz, 3H), 7.36 (d, *J* = 2.1 Hz, 1H), 7.28 − 7.21 (m, 2H), 5.31 (s, 2H), 4.52 (t, *J* = 5.5 Hz, 2H), 3.76 (q, *J* = 6.8, 6.1 Hz, 2H), 2.58 (s, 3H). ^13^C NMR (100 MHz, DMSO-d_6_) *δ* 175.7, 163.7, 163.3, 163.1, 159.2, 157.8, 143.3, 142.2, 139.0, 138.9, 133.7, 131.0, 128.6, 128.1, 127.5, 127.0, 117.9, 116.8, 115.3, 111.0, 102.5, 69.7, 55.6, 38.7, 13.1; ESI-HRMS: *m/z* calcd for C_25_H_21_ClN_2_O_6_ [M + H]^+^: 481.1161; found: 481.1167. HPLC purity: 99.7%.

#### 7-((4-Chlorobenzyl)oxy)-N-(2–(3-hydroxy-2-methyl-4-oxopyridin-1(4H)-yl)ethyl)-4-oxo-4H-chromene-3-carboxamide (15g)

Yield: 23%, yellow solid, m.p. = 241–243 °C; ^1^H NMR (400 MHz, DMSO-d_6_) *δ* 9.36 (t, *J* = 6.1 Hz, 1H), 8.91 (s, 1H), 8.07 (dd, *J* = 7.9, 3.9 Hz, 2H), 7.54 − 7.46 (m, 4H), 7.36 (d, *J* = 2.2 Hz, 1H), 7.26 − 7.18 (m, 2H), 5.30 (s, 2H), 4.52 (t, *J* = 5.7 Hz, 2H), 3.76 (q, *J* = 5.7 Hz, 2H), 2.58 (s, 3H). ^13^C NMR (100 MHz, DMSO-d_6_) *δ* 175.7, 163.8, 163.3, 163.0, 159.3, 157.9, 143.3, 142.1, 139.1, 135.4, 133.3, 130.3, 129.1, 127.5, 117.8, 116.9, 115.3, 110.9, 102.5, 69.9, 55.6, 38.7, 13.1; ESI-HRMS: *m/z* calcd for C_25_H_21_ClN_2_O_6_ [M + H]^+^: 481.1161; found: 481.1170. HPLC purity: 98.1%.

#### 3-Hydroxy-2-methyl-1–(2-(((4-oxo-4H-chromen-3-yl)methyl)amino)ethyl)pyridin-4(1H)-one (17a)

Yield: 92%, yellow solid, m.p. = 248–250 °C; ^1^H NMR (400 MHz, DMSO-d_6_) *δ* 9.81 (s, 2H), 8.72 (s, 1H), 8.28 (d, *J* = 7.0 Hz, 1H), 8.12 (dd, *J* = 8.0, 1.5 Hz, 1H), 7.88 (ddd, *J* = 8.7, 7.2, 1.7 Hz, 1H), 7.73 (d, *J* = 8.4 Hz, 1H), 7.59 − 7.55 (m, 1H), 7.31 (d, *J* = 6.9 Hz, 1H), 4.74 (t, *J* = 6.8 Hz, 2H), 4.05 (s, 2H), 3.45 (s, 2H), 2.56 (s, 3H). ^13^C NMR (100 MHz, DMSO-d_6_) δ 175.9, 159.3, 158.7, 155.9, 143.1, 142.1, 138.6, 134.9, 126.1, 125.1, 123.0, 118.7, 115.1, 111.0, 51.5, 45.0, 41.4, 13.0. ESI-HRMS: *m/z* calcd for C_18_H_18_N_2_O_4_ [M + H]^+^: 327.1339; found: 327.1332. HPLC purity: 99.7%.

#### 3-Hydroxy-2-methyl-1–(2-(((7-methyl-4-oxo-4H-chromen-3-yl)methyl)amino)ethyl)pyridin-4(1H)-one (17b)

Yield: 92.9%, yellow solid, m.p. = 251–253 °C; ^1^H NMR (400 MHz, DMSO-d_6_) *δ* 9.80 (s, 2H), 8.69 − 8.64 (m, 1H), 8.32 − 8.24 (m, 1H), 7.99 (d, *J* = 8.1 Hz, 1H), 7.55 (s, 1H), 7.36 (dd, *J* = 24.6, 8.2 Hz, 2H), 4.78 − 4.68 (m, 2H), 4.03 (s, 2H), 3.44 (s, 2H), 2.56 (s, 3H), 2.48 (s, 3H). ^13^C NMR (100 MHz, DMSO-d_6_) *δ* 176.2, 159.7, 158.9, 156.5, 146.5, 143.5, 142.4, 139.1, 127.9, 125.3, 121.3, 118.6, 115.4, 111.4, 52.0, 45.5, 42.0, 21.7, 13.4. ESI-HRMS: *m/z* calcd for C_19_H_20_N_2_O_4_ [M + H]^+^: 341.1496; found: 341.1490. HPLC purity: 99.1%.

#### 3-Hydroxy-2-methyl-1–(2-(((6-methyl-4-oxo-4H-chromen-3-yl)methyl)amino) ethyl)pyridin-4(1H)-one (17c)

Yield: 77.8%, yellow solid, m.p. = 259–261 °C; ^1^H NMR (400 MHz, DMSO-d_6_) *δ* 9.87 (s, 2H), 8.69 (s, 1H), 8.29 (d, *J* = 7.0 Hz, 1H), 7.89 (s, 1H), 7.69 (dd, *J* = 8.6, 2.0 Hz, 1H), 7.62 (d, *J* = 8.6 Hz, 1H), 7.32 (d, *J* = 7.0 Hz, 1H), 4.74 (t, *J* = 6.7 Hz, 2H), 4.04 (s, 2H), 3.44 (t, *J* = 5.9 Hz, 2H), 2.56 (s, 3H), 2.45 (s, 3H); ^13^C NMR (100 MHz, DMSO-d_6_) *δ* 176.4, 159.8, 159.0, 154.7, 143.6, 142.3, 139.1, 136.4, 136.3, 124.7, 123.2, 118.9, 115.3, 111.4, 51.9, 45.5, 42.0, 20.9, 13.4. ESI-HRMS: *m/z* calcd for C_19_H_20_N_2_O_4_ [M + H]^+^: 341.1496; found: 341.1490. HPLC purity: 99.2%.

#### 3-Hydroxy-1–(2-(((7-methoxy-4-oxo-4H-chromen-3-yl)methyl)amino)ethyl)-2-methylpyridin-4(1H)-one (17d)

Yield: 89.6%, yellow solid, m.p. = 252–254 °C; ^1^H NMR (400 MHz, DMSO-d_6_) *δ* 9.74 (s, 2H), 8.63 (s, 1H), 8.28 (d, *J* = 7.0 Hz, 1H), 8.01 (d, *J* = 8.9 Hz, 1H), 7.31 (d, *J* = 7.0 Hz, 1H), 7.22 (d, *J* = 2.3 Hz, 1H), 7.13 (dd, *J* = 8.9, 2.3 Hz, 1H), 4.73 (t, *J* = 6.7 Hz, 2H), 4.02 (s, 2H), 3.91 (s, 3H), 3.44 (s, 2H), 2.56 (s, 3H); ^13^C NMR (100 MHz, DMSO-d_6_) δ 175.6, 164.7, 159.8, 158.6, 158.3, 143.6, 142.4, 139.1, 127.0, 117.2, 115.8, 115.3, 111.4, 101.4, 56.8, 52.0, 45.5, 42.0, 13.4. ESI-HRMS: *m/z* calcd for C_19_H_20_N_2_O_5_ [M + H]^+^: 357.1445; found: 357.1460. HPLC purity: 97.3%.

#### 3-Hydroxy-1–(2-(((6-methoxy-4-oxo-4H-chromen-3-yl)methyl)amino)ethyl)-2-methylpyridin-4(1H)-one (17e)

Yield: 90.6%, yellow solid, m.p. = 267–269 °C; ^1^H NMR (400 MHz, DMSO-d_6_) *δ* 9.89 (s, 2H), 8.71 (s, 1H), 8.31 (d, *J* = 7.0 Hz, 1H), 7.70 (d, *J* = 9.1 Hz, 1H), 7.53 − 7.41 (m, 2H), 7.36 (d, *J* = 7.0 Hz, 1H), 4.75 (t, *J* = 6.6 Hz, 2H), 4.05 (s, 2H), 3.87 (s, 3H), 3.45 (s, 2H), 2.56 (s, 3H). ^13^C NMR (100 MHz, DMSO-d_6_) *δ* 175.7, 159.3, 158.5, 157.0, 150.7, 143.1, 142.0, 138.7, 123.9, 123.8, 120.4, 114.2, 111.0, 104.8, 55.9, 51.6, 45.1, 41.5, 13.0. ESI-HRMS: *m/z* calcd for C_19_H_20_N_2_O_5_ [M + H]^+^: 357.1445; found: 357.1448. HPLC purity: 99.3%.

#### 1–(2-(((7-(Benzyloxy)-4-oxo-4H-chromen-3-yl)methyl)amino)ethyl)-3-hydroxy-2-methylpyridin-4(1H)-one (17f)

Yield: 54.4%, yellow solid, m.p. = 169–171 °C; ^1^H NMR (400 MHz, DMSO-d_6_) *δ* 9.73 (s, 2H), 8.62 (s, 1H), 8.27 (d, *J* = 7.0 Hz, 1H), 8.02 (d, *J* = 8.9 Hz, 1H), 7.49 (d, *J* = 7.0 Hz, 2H), 7.44 − 7.27 (m, 5H), 7.21 (dd, *J* = 8.9, 2.3 Hz, 1H), 5.29 (s, 2H), 4.72 (t, *J* = 6.6 Hz, 2H), 4.02 (s, 2H), 3.43 (s, 2H), 2.55 (s, 3H); ^13^C NMR (100 MHz, DMSO-d_6_) δ 175.6, 163.7, 159.8, 158.6, 158.2, 143.6, 142.4, 139.1, 136.4, 129.0, 128.7, 128.4, 127.0, 117.4, 116.3, 115.3, 111.4, 102.4, 70.7, 52.0, 45.5, 42.0, 13.4. ESI-HRMS: *m/z* calcd for C_25_H_24_N_2_O_5_ [M + H]^+^: 433.1758; found: 433.1769. HPLC purity: 98.3%.

#### 3-Hydroxy-2-methyl-1–(2-(((7-((3-methylbenzyl)oxy)-4-oxo-4H-chromen-3-yl)methyl)amino)ethyl)pyridin-4(1H)-one (17g)

Yield: 35.9%, yellow solid, m.p. = 211–213 °C; ^1^H NMR (400 MHz, DMSO-d_6_) *δ* 9.72 (s, 2H), 8.62 (s, 1H), 8.25 (d, *J* = 7.0 Hz, 1H), 8.02 (d, *J* = 8.9 Hz, 1H), 7.32 − 7.25 (m, 5H), 7.22 − 7.16 (m, 2H), 5.24 (s, 2H), 4.71 (t, *J* = 6.5 Hz, 2H), 4.02 (s, 2H), 3.43 (s, 2H), 2.55 (s, 3H), 2.33 (s, 3H); ^13^C NMR (100 MHz, DMSO-d_6_) δ 175.6, 163.7, 160.1, 158.6, 158.2, 143.6, 142.0, 139.1, 138.2, 136.3, 129.3, 129.0, 128.9, 127.0, 125.5, 117.3, 116.3, 115.3, 111.5, 102.4, 70.7, 51.9, 45.5, 42.0, 21.5, 13.4. ESI-HRMS: *m/z* calcd for C_26_H_26_N_2_O_5_ [M + H]^+^: 447.1914; found: 447.1934. HPLC purity: 99.7%.

#### 3-Hydroxy-2-methyl-1–(2-(((7-((4-methylbenzyl)oxy)-4-oxo-4H-chromen-3-yl)methyl)amino)ethyl)pyridin-4(1H)-one (17h)

Yield: 90.1%, yellow solid, m.p. = 134–136 °C; ^1^H NMR (400 MHz, DMSO-d_6_) *δ* 9.64 (s, 2H), 8.60 (s, 1H), 8.22 (d, *J* = 5.6 Hz, 1H), 8.01 (d, *J* = 8.9 Hz, 1H), 7.37 (d, *J* = 8.0 Hz, 2H), 7.30 (d, *J* = 2.3 Hz, 1H), 7.24 − 7.17 (m, 4H), 5.23 (s, 2H), 4.69 (t, *J* = 5.7 Hz, 2H), 4.02 (s, 2H), 3.43 (s, 2H), 2.54 (s, 3H), 2.31 (s, 3H); ^13^C NMR (100 MHz, DMSO-d_6_) δ 175.6, 163.7, 160.0, 158.6, 158.2, 143.6, 142.1, 139.1, 138.0, 133.4, 129.6, 128.6, 127.0, 117.3, 116.3, 115.3, 111.5, 102.4, 70.6, 51.9, 45.5, 41.9, 21.3, 13.4. ESI-HRMS: *m/z* calcd for C_26_H_26_N_2_O_5_ [M + H]^+^: 447.1914; found: 447.1908. HPLC purity: 98%.

#### 1–(2-(((7-((3-Fluorobenzyl)oxy)-4-oxo-4H-chromen-3-yl)methyl)amino)ethyl)-3-hydroxy-2-methylpyridin-4(1H)-one (17i)

Yield: 62.8%, yellow solid, m.p. = 215–217 °C; ^1^H NMR (400 MHz, DMSO-d_6_) *δ* 9.78 (s, 2H), 8.63 (s, 1H), 8.27 (d, *J* = 7.0 Hz, 1H), 8.03 (d, *J* = 8.9 Hz, 1H), 7.47 (q, *J* = 7.6 Hz, 1H), 7.35 − 7.28 (m, 4H), 7.20 (ddd, *J* = 14.5, 8.2, 2.0 Hz, 2H), 5.32 (s, 2H), 4.73 (t, *J* = 6.7 Hz, 2H), 4.02 (s, 2H), 3.43 (s, 2H), 2.55 (s, 3H); ^13^C NMR (100 MHz, DMSO-d_6_) δ 175.2, 163.0, 162.2 (d, ^1^*J* = 243 Hz), 159.5, 158.2, 157.7, 143.2, 141.8, 138.9 (d, ^3^*J* = 7 Hz), 138.7, 130.7 (d, ^3^*J* = 9 Hz), 126.7, 123.9 (d, ^4^*J* = 3 Hz), 117.1, 115.9, 115.0 (d, ^2^*J* = 21 Hz), 114.9, 114.6 (d, ^2^*J* = 22 Hz), 111.0, 102.1, 69.3, 51.5, 45.1, 41.6, 13.0. ESI-HRMS: *m/z* calcd for C_25_H_23_FN_2_O_5_ [M + H]^+^: 451.1664; found: 451.1676. HPLC purity: 97.9%.

#### 1–(2-(((7-((4-Fluorobenzyl)oxy)-4-oxo-4H-chromen-3-yl)methyl)amino)ethyl)-3-hydroxy-2-methylpyridin-4(1H)-one (17j)

Yield: 43.5%, yellow solid, m.p. = 218–220 °C; ^1^H NMR (400 MHz, DMSO-d_6_) *δ* 9.74 (s, 2H), 8.62 (s, 1H), 8.26 (d, *J* = 7.0 Hz, 1H), 8.02 (d, *J* = 8.9 Hz, 1H), 7.55 (dd, *J* = 8.4, 5.6 Hz, 2H), 7.32 (d, *J* = 2.2 Hz, 1H), 7.24 (ddd, *J* = 21.6, 7.9, 6.0 Hz, 4H), 5.27 (s, 2H), 4.72 (t, *J* = 6.6 Hz, 2H), 4.02 (s, 2H), 3.44 (s, 2H), 2.55 (s, 3H); ^13^C NMR (100 MHz, DMSO-d_6_) δ175.6, 163.5, 162.4 (d, ^1^*J* = 242 Hz), 159.6, 158.6, 158.1, 143.5, 142.6, 139.1, 132.7 (d, ^4^*J* = 3 Hz), 130.8 (d, ^3^*J* = 8 Hz), 127.1, 117.4, 116.3, 115.9 (d, ^2^*J* = 21 Hz), 115.3, 111.4, 102.4, 69.9, 52.0, 45.5, 42.0, 13.4. ESI-HRMS: *m/z* calcd for C_25_H_23_FN_2_O_5_ [M + H]^+^: 451.1664; found: 451.1683. HPLC purity: 98%.

#### 1–(2-(((7-((3-Chlorobenzyl)oxy)-4-oxo-4H-chromen-3-yl)methyl)amino)ethyl)-3-hydroxy-2-methylpyridin-4(1H)-one (17k)

Yield: 63.9%, yellow solid, m.p. = 226–228 °C; ^1^H NMR (400 MHz, DMSO-d_6_) *δ* 9.63 (s, 2H), 8.61 (s, 1H), 8.22 (d, *J* = 7.0 Hz, 1H), 8.03 (d, *J* = 8.9 Hz, 1H), 7.57 (s, 1H), 7.47 − 7.42 (m, 3H), 7.32 (d, *J* = 2.3 Hz, 1H), 7.24 − 7.20 (m, 2H), 5.31 (s, 2H), 4.69 (t, *J* = 6.8 Hz, 2H), 4.02 (s, 2H), 3.43 (s, 2H), 2.54 (s, 3H); ^13^C NMR (100 MHz, DMSO-d_6_) δ 175.6, 163.4, 160.2, 158.6, 158.1, 143.7, 141.9, 139.1, 139.0, 133.7, 131.0, 128.6, 128.1, 127.1, 126.9, 117.5, 116.3, 115.3, 111.5, 102.5, 69.6, 51.9, 45.6, 42.0, 13.3. ESI-HRMS: *m/z* calcd for C_25_H_23_ClN_2_O_5_ [M + H]^+^: 467.1368; found: 467.1388. HPLC purity: 99.7%.

#### 1–(2-(((7-((4-Chlorobenzyl)oxy)-4-oxo-4H-chromen-3-yl)methyl)amino)ethyl)-3-hydroxy-2-methylpyridin-4(1H)-one (17l)

Yield: 65%, yellow solid, m.p. = 189–191 °C; ^1^H NMR (400 MHz, DMSO-d_6_) *δ* 9.68 (s, 2H), 8.61 (s, 1H), 8.25 (d, *J* = 7.0 Hz, 1H), 8.02 (d, *J* = 8.9 Hz, 1H), 7.52 (d, *J* = 8.5 Hz, 2H), 7.48 (d, *J* = 8.5 Hz, 2H), 7.31 (d, *J* = 2.3 Hz, 1H), 7.25 (d, *J* = 6.8 Hz, 1H), 7.21 (dd, *J* = 8.9, 2.4 Hz, 1H), 5.29 (s, 2H), 4.71 (t, *J* = 6.7 Hz, 2H), 4.02 (s, 2H), 3.43 (s, 2H), 2.55 (s, 3H); ^13^C NMR (100 MHz, DMSO-d_6_) δ 175.6, 163.5, 160.0, 158.6, 158.1, 143.6, 142.1, 139.1, 135.5, 133.3, 130.3, 129.1, 127.1, 117.5, 116.3, 115.3, 111.5, 102.5, 69.8, 51.9, 45.5, 42.0, 13.4. ESI-HRMS: *m/z* calcd for C_25_H_23_ClN_2_O_5_ [M + H]^+^: 467.1368; found: 467.1363. HPLC purity: 99.7%.

### General procedure for the preparation of compounds 10 and 11

The synthesis procedure of compound **10** was the same as that of compounds **5a–l**. A mixture of compound **10** (10 mmol), and triethylamine (20 mmol) in anhydrous DCM (80 ml) was cooled to 0 °C for a few minutes. Then acetyl chloride (10 mmol) dissolved in anhydrous DCM was added dropwise. After the addition was finished, the reaction mixture was stirred for 3 h at room temperature. The reaction solution was washed by water three times, evaporated under vacuum, and purified by column chromatography hexane/ethyl acetate. The target product **11** was obtained by removing the solvent *in vacuo*.

#### 7-Hydroxy-4-oxo-4H-chromene-3-carbaldehyde (10)

Yield: 51%, Rufous solid, m.p. = 265–267 °C; ^1^H NMR (400 MHz, DMSO-d_6_) δ 11.07 (s, 1H), 10.10 (s, 1H), 8.76 (s, 1H), 7.96 (d, *J* = 8.8 Hz, 1H), 6.98 (d, *J* = 8.8 Hz, 1H), 6.93 (s, 1H); ^13^C NMR (100 MHz, DMSO-d_6_) *δ* 188.7, 174.1, 163.5, 162.7, 157.4, 127.1, 119.7, 117.0, 116.0, 103.1.

#### 3*-Formyl-4-oxo-4H-chromen-7-yl acetate (11)*

Yield: 54.3%, yellow solid, m.p. = 168–170 °C; ^1^H NMR (400 MHz, CDCl_3_) *δ* 10.40 (s, 1H), 8.56 (s, 1H), 8.34 (d, *J* = 8.7 Hz, 1H), 7.40 (d, *J* = 1.9 Hz, 1H), 7.27 (d, *J* = 2.0 Hz, 1H), 2.40 (s, 3H). ^13^C NMR (100 MHz, CDCl_3_) *δ* 189.1, 175.4, 165.0, 160.4, 158.1, 127.6, 120.3, 118.9, 115.7, 101.2, 56.2.

### General procedure for the preparation of compounds 12 and 13

The synthesis procedure of compound **12** was the same as that of compounds **6a–e**. A solution of intermediate **12** (1.0 mmol) in anhydrous CH_2_Cl_2_ (10 ml) was treated with DMF (2 drops). After stirring for a few minutes at room temperature, oxalyl chloride (2.0 mmol) was added dropwise to the above system. For the generation of corresponding acyl chloride, the mixture was allowed to stir for further 3 h. Subsequently, the reaction solvent was evaporated, dissolved by anhydrous DCM renewedly, and added dropwise to a solution of the intermediate **3** (2 mmol) and triethylamine (4 mmol) in anhydrous CH_2_Cl_2_ at 0 °C. After stirring for another 3 h at room temperature, the crude product was afforded by washed with water three times, dried, and concentrated *in vacuo*. The target compound was purified by column chromatography DCM/MeOH; the solvents were concentrated to give **13**.

#### 7-Acetoxy-4-oxo-4H-chromene-3-carboxylic acid (12)

Yield: 44.3%, yellow solid, m.p. = 202–204 °C; ^1^H NMR (400 MHz, DMSO-d_6_) *δ* 13.23 (s, 1H), 9.09 (s, 1H), 8.19 (d, *J* = 8.7 Hz, 1H), 7.69 (d, *J* = 2.0 Hz, 1H), 7.41 (dd, *J* = 8.7, 2.1 Hz, 1H), 2.34 (s, 3H); ^13^C NMR (100 MHz, DMSO-d_6_) *δ* 176.6, 165.5, 164.4, 164.2, 158.4, 127.3, 117.0, 116.8, 114.0, 101.7, 57.0.

#### 3-((2–(3-((4-Methoxybenzyl)oxy)-2-methyl-4-oxopyridin-1(4H)-yl)ethyl)carbamoyl)-4-oxo-4H-chromen-7-yl acetate (13)

Yield: 23.6%, yellow oil, ^1^H NMR (400 MHz, CDCl_3_) *δ* 9.48 (t, *J* = 5.8 Hz, 1H), 8.91 (s, 1H), 8.28 (d, *J* = 8.8 Hz, 1H), 7.41 (d, *J* = 2.0 Hz, 1H), 7.32 (d, *J* = 8.6 Hz, 2H), 7.28 (d, *J* = 2.1 Hz, 1H), 7.23 (d, *J* = 7.5 Hz, 1H), 6.85 (d, *J* = 8.6 Hz, 2H), 6.44 (d, *J* = 7.5 Hz, 1H), 5.15 (s, 2H), 4.02 (t, *J* = 6.6 Hz, 2H), 3.78 (s, 3H), 3.61 (q, *J* = 6.4 Hz, 2H), 2.37 (s, 3H), 2.14 (s, 3H). ^13^C NMR (100 MHz, CDCl_3_) *δ* 176.4, 173.4, 165.0, 164.0, 162.0, 159.5, 158.0, 146.0, 141.3, 138.6, 130.9, 129.7, 127.6, 117.7, 117.3, 116.0, 114.9, 113.6, 100.5, 72.8, 56.1, 55.3, 52.3, 39.4, 12.6.

### General procedure for the preparation of compounds 14a–g

Potassium carbonate (1.4 mmol) was added to the solution of intermediate **13** (1 mmol) in component solvent (acetone: water = 1: 1, 10 ml). After refluxing for 30 min, the ester group of intermediate **13** was hydrolysed. Then the mixture was evaporated and neutralised by adding 1 N HCl dropwise till no more solids precipitated. Filtered, washed with water to yield a faint yellow solid. Subsequently, appropriate benzyl bromide (1.2 eq) and K_2_CO_3_ (1.3 eq) were added to a solution of above intermediate (1 mmol) in DMF (4 ml). After stirring at 80 °C for another 12 h, the mixture was poured into water and extracted with dichloromethane three times, and washed with water. The collected organic layers were removed under vacuum to obtain yellow oil, which was purified by chromatography (DCM: MeOH = 30:1) to obtain the final compounds.

#### 7-(Benzyloxy)-N-(2–(3-((4-methoxybenzyl)oxy)-2-methyl-4-oxopyridin-1(4H)-yl)ethyl)-4-oxo-4H-chromene-3-carboxamide (14a)

Yield: 25%, yellow oil, ^1^H NMR (400 MHz, CDCl_3_) *δ* 9.61 (t, *J* = 5.8 Hz, 1H), 8.83 (s, 1H), 8.15 (d, *J* = 9.0 Hz, 1H), 7.43 (t, *J* = 6.3 Hz, 5H), 7.32 (d, *J* = 8.5 Hz, 2H), 7.20 (d, *J* = 7.5 Hz, 1H), 7.13 (dd, *J* = 9.0, 2.2 Hz, 1H), 7.00 (d, *J* = 2.1 Hz, 1H), 6.84 (d, *J* = 8.5 Hz, 2H), 6.40 (d, *J* = 7.5 Hz, 1H), 5.18 (s, 2H), 5.14 (s, 2H), 3.99 (t, *J* = 6.6 Hz, 2H), 3.78 (s, 3H), 3.60 (q, *J* = 6.4 Hz, 2H), 2.14 (s, 3H); ^13^C NMR (100 MHz, CDCl_3_) *δ* 176.3, 173.4, 164.1, 164.0, 162.0, 159.5, 157.9, 146.0, 141.2, 138.5, 135.2, 130.9, 129.7, 128.9, 128.6, 127.7, 127.6, 117.9, 117.3, 116.5, 114.9, 113.6, 101.7, 72.8, 70.9, 55.3, 52.3, 39.4, 12.6; ESI-HRMS: *m/z* calcd for C_33_H_30_N_2_O_7_ [M + H]^+^: 567.2126; found: 567.2111.

#### N-(2–(3-((4-methoxybenzyl)oxy)-2-methyl-4-oxopyridin-1(4H)-yl)ethyl)-7-((3-methylbenzyl)oxy)-4-oxo-4H-chromene-3-carboxamide (14b)

Yield: 15%, yellow oil, ^1^H NMR (400 MHz, DMSO-d_6_) *δ* 9.34 (t, *J* = 6.0 Hz, 1H), 8.93 (s, 1H), 8.05 (d, *J* = 8.9 Hz, 1H), 7.51 (d, *J* = 7.5 Hz, 1H), 7.32 (d, *J* = 2.3 Hz, 1H), 7.28 (q, *J* = 7.3, 4.9 Hz, 5H), 7.21 (dd, *J* = 9.0, 2.3 Hz, 1H), 7.16 (d, *J* = 6.7 Hz, 1H), 6.87 (d, *J* = 8.6 Hz, 2H), 6.11 (d, *J* = 7.5 Hz, 1H), 5.22 (s, 2H), 4.92 (s, 2H), 4.05 (t, *J* = 6.3 Hz, 2H), 3.72 (s, 3H), 3.58 (q, J = 6.2 Hz, 2H), 2.32 (s, 3H), 2.19 (s, 3H). ^13^C NMR (100 MHz, DMSO-d_6_) *δ* 175.8, 172.5, 164.0, 163.1, 163.0, 159.4, 157.9, 145.7, 141.3, 140.0, 138.3, 136.2, 130.6, 130.2, 129.3, 129.0, 128.9, 127.4, 125.6, 117.7, 116.8, 116.5, 115.4, 114.0, 102.4, 72.0, 70.8, 55.5, 52.1, 39.2, 21.4, 12.5.

#### N-(2–(3-((4-methoxybenzyl)oxy)-2-methyl-4-oxopyridin-1(4H)-yl)ethyl)-7-((4-methylbenzyl)oxy)-4-oxo-4H-chromene-3-carboxamide (14c)

Yield: 16%, yellow oil, ^1^H NMR (400 MHz, DMSO-d_6_) *δ* 9.34 (t, *J* = 6.0 Hz, 1H), 8.93 (s, 1H), 8.05 (d, *J* = 8.9 Hz, 1H), 7.54 (d, *J* = 7.5 Hz, 1H), 7.37 (d, *J* = 7.9 Hz, 2H), 7.33 − 7.28 (m, 3H), 7.23 − 7.18 (m, 3H), 6.87 (d, *J* = 8.6 Hz, 2H), 6.15 (d, *J* = 7.5 Hz, 1H), 5.22 (s, 2H), 4.92 (s, 2H), 4.07 (t, *J* = 6.3 Hz, 2H), 3.72 (s, 3H), 3.58 (q, *J* = 6.1 Hz, 2H), 2.30 (s, 3H), 2.21 (s, 3H); ^13^C NMR (100 MHz, DMSO-d_6_) δ 175.8, 172.1, 164.0, 163.1, 163.0, 159.4, 157.9, 145.6, 141.8, 140.1, 138.0, 133.3, 130.6, 130.1, 129.6, 128.6, 127.4, 117.7, 116.9, 116.3, 115.4, 114.0, 102.4, 72.1, 70.7, 55.5, 52.3, 39.2, 21.3, 12.5.

#### 7-((3-Fluorobenzyl)oxy)-N-(2–(3-((4-methoxybenzyl)oxy)-2-methyl-4-oxopyridin-1(4H)-yl)ethyl)-4-oxo-4H-chromene-3-carboxamide (14d)

Yield: 19%, yellow oil, ^1^H NMR (400 MHz, CDCl_3_) *δ* 9.61 (t, *J* = 5.9 Hz, 1H), 8.83 (s, 1H), 8.17 (d, *J* = 9.0 Hz, 1H), 7.42 − 7.28 (m, 4H), 7.21 (d, *J* = 7.7 Hz, 1H), 7.18 − 7.12 (m, 2H), 7.05 (dd, *J* = 8.5, 2.2 Hz, 1H), 6.99 (d, *J* = 2.3 Hz, 1H), 6.85 (d, *J* = 8.6 Hz, 2H), 6.58 (s, 1H), 5.18 (s, 2H), 5.16 (s, 2H), 4.05 (t, *J* = 6.4 Hz, 2H), 3.79 (s, 3H), 3.63 (q, *J* = 6.3 Hz, 2H), 2.18 (s, 3H); ^13^C NMR (100 MHz, CDCl_3_) *δ* 176.4, 173.4, 164.0, 163.8, 163.1 (d, ^1^*J* = 245 Hz), 162.1, 159.6, 158.0, 146.1, 141.2, 138.6, 137.9 (d, ^3^*J* = 7 Hz), 131.0, 130.6 (d, ^3^*J* = 9 Hz), 129.8, 127.9, 122.9 (d, ^4^*J* = 3 Hz), 118.2, 117.4, 116.4, 115.6 (d, ^2^*J* = 21 Hz), 115.1, 114.4 (d, ^2^*J* = 22 Hz), 113.7, 101.8, 72.8, 70.0, 55.4, 52.3, 39.5, 12.7.

#### 7-((4-Fluorobenzyl)oxy)-N-(2–(3-((4-methoxybenzyl)oxy)-2-methyl-4-oxopyridin-1(4H)-yl)ethyl)-4-oxo-4H-chromene-3-carboxamide (14e)

Yield: 15%, yellow oil, ^1^H NMR (400 MHz, CDCl_3_) *δ* 9.59 (t, *J* = 5.9 Hz, 1H), 8.83 (s, 1H), 8.15 (d, *J* = 9.0 Hz, 1H), 7.41 (dd, *J* = 8.5, 5.3 Hz, 2H), 7.31 (d, *J* = 8.5 Hz, 2H), 7.22 (d, *J* = 7.5 Hz, 1H), 7.10 (td, *J* = 8.9, 2.2 Hz, 3H), 6.98 (d, *J* = 2.3 Hz, 1H), 6.84 (d, *J* = 8.6 Hz, 2H), 6.41 (d, *J* = 7.5 Hz, 1H), 5.13 (s, 4H), 4.00 (t, *J* = 6.6 Hz, 2H), 3.77 (s, 3H), 3.60 (q, *J* = 6.4 Hz, 2H), 2.14 (s, 3H); ^13^C NMR (100 MHz, CDCl_3_) *δ* 176.4, 173.4, 164.0, 163.9, 162.9 (d, ^1^*J* = 246 Hz), 162.1, 159.6, 158.0, 146.1, 141.2, 138.6, 131.1 (d, ^4^*J* = 3 Hz), 130.9, 129.8, 129.6 (d, ^3^*J* = 8 Hz), 127.8, 118.1, 117.4, 116.5, 115.9 (d, ^2^*J* = 21 Hz), 115.1, 113.7, 101.7, 72.8, 70.2, 55.4, 52.3, 39.5, 12.7.

#### 7-((3-Chlorobenzyl)oxy)-N-(2–(3-((4-methoxybenzyl)oxy)-2-methyl-4-oxopyridin-1(4H)-yl)ethyl)-4-oxo-4H-chromene-3-carboxamide (14f)

Yield: 18%, yellow oil, ^1^H NMR (400 MHz, CDCl_3_) *δ* 9.62 (t, *J* = 5.8 Hz, 1H), 8.85 (s, 1H), 8.18 (d, *J* = 9.0 Hz, 1H), 7.45 (s, 1H), 7.37 − 7.31 (m, 5H), 7.21 (d, *J* = 7.5 Hz, 1H), 7.14 (dd, *J* = 9.0, 2.3 Hz, 1H), 6.99 (d, *J* = 2.3 Hz, 1H), 6.85 (d, *J* = 8.6 Hz, 2H), 6.41 (d, *J* = 7.4 Hz, 1H), 5.16 (s, 2H), 5.14 (s, 2H), 4.00 (t, *J* = 6.6 Hz, 2H), 3.79 (s, 3H), 3.60 (q, *J* = 6.4 Hz, 2H), 2.13 (s, 3H); ^13^C NMR (100 MHz, CDCl_3_) *δ* 176.3, 173.3, 164.0, 163.7, 162.1, 159.5, 157.9, 145.9, 141.5, 138.7, 137.3, 134.8, 130.8, 130.2, 129.6, 128.7, 127.8, 127.5, 125.4, 118.1, 117.1, 116.3, 115.0, 113.6, 101.7, 72.8, 69.9, 55.3, 52.4, 39.4, 12.6.

#### 7-((4-Chlorobenzyl)oxy)-N-(2–(3-((4-methoxybenzyl)oxy)-2-methyl-4-oxopyridin-1(4H)-yl)ethyl)-4-oxo-4H-chromene-3-carboxamide (14g)

Yield: 16%, yellow oil, ^1^H NMR (600 MHz, DMSO-d_6_) *δ* 9.33 (t, *J* = 6.1 Hz, 1H), 8.95 (s, 1H), 8.08 (d, *J* = 8.9 Hz, 1H), 7.54 − 7.47 (m, 5H), 7.36 (d, *J* = 2.3 Hz, 1H), 7.30 (d, *J* = 8.5 Hz, 2H), 7.24 (dd, *J* = 8.9, 2.3 Hz, 1H), 6.88 (d, *J* = 8.5 Hz, 2H), 6.12 (d, *J* = 7.5 Hz, 1H), 5.30 (s, 2H), 4.92 (s, 2H), 4.06 (t, *J* = 6.4 Hz, 2H), 3.73 (s, 3H), 3.59 (q, *J* = 6.3 Hz, 2H), 2.20 (s, 3H). ^13^C NMR (150 MHz, DMSO-d_6_) *δ* 175.4, 172.0, 163.4, 162.6, 162.6, 159.0, 157.4, 145.2, 140.9, 139.5, 135.0, 132.9, 130.1, 129.8, 129.8, 128.6, 127.0, 117.4, 116.4, 116.0, 115.0, 113.5, 102.0, 71.6, 69.4, 55.0, 51.6, 40.1, 12.0.

### General procedure for the preparation of compounds 16a–l

To a solution of compounds **5a–l** (1 mmol) in anhydrous DCM (10 ml), Na(AcO)_3_BH (1.4 mmol) and intermediate **3** (1 mmol) were added under argon atmosphere at room temperature. After stirring for 6 h, the mixture was diluted with CH_2_Cl_2_ and washed with saturated NaHCO_3_ solution. The collected organic phases were dried over anhydrous sodium sulphate and removed *in vacuo*. The residue was purified by flash chromatography DCM/MeOH to afford intermediates **16a–l**.

#### 3-((4-Methoxybenzyl)oxy)-2-methyl-1–(2-(((4-oxo-4H-chromen-3-yl)methyl) amino)ethyl)pyridin-4(1H)-one (16a)

Yield: 30%, yellow oil, ^1^H NMR (400 MHz, CDCl_3_) *δ* 8.15 (dd, *J* = 8.0, 1.4 Hz, 1H), 7.82 (s, 1H), 7.67 − 7.61 (m, 1H), 7.43 − 7.35 (m, 2H), 7.28 (s, 2H), 7.25 (s, 1H), 6.78 (d, *J* = 8.5 Hz, 2H), 6.35 (d, *J* = 7.5 Hz, 1H), 5.09 (s, 2H), 3.83 (t, *J* = 6.3 Hz, 2H), 3.72 (s, 3H), 3.54 (s, 2H), 2.79 (t, *J* = 6.3 Hz, 2H), 2.05 (s, 3H). ^13^C NMR (100 MHz, CDCl_3_) *δ* 178.0, 173.4, 159.4, 156.5, 153.5, 145.8, 141.0, 138.9, 133.9, 130.8, 129.7, 125.6, 125.3, 123.7, 121.6, 118.3, 116.9, 113.6, 72.5, 55.2, 53.6, 48.6, 45.5, 12.6.

#### 3-((4-Methoxybenzyl)oxy)-2-methyl-1–(2-(((7-methyl-4-oxo-4H-chromen-3-yl)methyl)amino)ethyl)pyridin-4(1H)-one (16b)

Yield: 27%, yellow oil, ^1^H NMR (400 MHz, CDCl_3_) *δ* 8.05 (dd, *J* = 8.4, 3.3 Hz, 1H), 7.79 (d, *J* = 9.1 Hz, 1H), 7.28 (d, *J* = 8.5 Hz, 2H), 7.22 (dt, *J* = 8.1, 4.9 Hz, 3H), 6.80 (d, *J* = 8.6 Hz, 2H), 6.37 (s, 1H), 5.11 (s, 2H), 3.84 (t, *J* = 6.3 Hz, 2H), 3.75 (s, 3H), 3.54 (s, 2H), 2.81 (t, *J* = 6.3 Hz, 2H), 2.46 (s, 3H), 2.06 (s, 3H); ^13^C NMR (100 MHz, CDCl_3_) δ 178.0, 173.5, 159.4, 156.7, 153.3, 145.8, 145.4, 141.1, 139.0, 130.8, 129.7, 126.9, 125.3, 121.5, 121.3, 117.9, 116.9, 113.6, 72.6, 55.2, 53.7, 48.6, 45.6, 21.9, 12.6.

#### 3-((4-Methoxybenzyl)oxy)-2-methyl-1–(2-(((6-methyl-4-oxo-4H-chromen-3-yl)methyl)amino)ethyl)pyridin-4(1H)-one (16c)

Yield: 28%, yellow oil, ^1^H NMR (400 MHz, CDCl_3_) *δ* 7.94 (s, 1H), 7.82 (s, 1H), 7.46 (d, *J* = 7.9 Hz, 1H), 7.30 (d, *J* = 26.9 Hz, 4H), 6.79 (d, *J* = 6.6 Hz, 2H), 6.36 (d, *J* = 5.5 Hz, 1H), 5.11 (s, 2H), 3.85 (s, 2H), 3.74 (s, 3H), 3.55 (s, 2H), 2.81 (s, 2H), 2.43 (s, 3H), 2.06 (s, 3H); ^13^C NMR (100 MHz, CDCl_3_) δ 178.1, 173.5, 159.4, 154.8, 153.6, 145.8, 141.1, 139.0, 135.4, 135.3, 130.9, 129.6, 124.9, 123.4, 121.0, 118.0, 117.0, 113.5, 72.6, 55.2, 53.5, 48.5, 45.5, 21.0, 12.7.

#### 1–(2-(((7-Methoxy-4-oxo-4H-chromen-3-yl)methyl)amino)ethyl)-3-((4-methoxybenzyl)oxy)-2-methylpyridin-4(1H)-one (16d)

Yield: 24%, yellow oil, ^1^H NMR (400 MHz, CDCl_3_) *δ* 8.07 (d, *J* = 8.9 Hz, 1H), 7.74 (d, *J* = 6.5 Hz, 1H), 7.29 (d, *J* = 8.5 Hz, 3H), 6.96 (dd, *J* = 8.9, 2.3 Hz, 1H), 6.80 (d, *J* = 8.7 Hz, 3H), 6.38 (d, *J* = 7.4 Hz, 1H), 5.12 (s, 2H), 3.88 (s, 3H), 3.86 − 3.82 (m, 2H), 3.75 (s, 3H), 3.53 (s, 2H), 2.80 (t, *J* = 6.3 Hz, 2H), 2.06 (s, 3H); ^13^C NMR (100 MHz, CDCl_3_) *δ* 177.3, 173.4, 164.2, 159.4, 158.3, 153.1, 145.8, 141.2, 139.1, 130.7, 129.6, 127.0, 121.3, 117.6, 116.9, 114.9, 113.6, 100.2, 72.6, 55.9, 55.2, 53.6, 48.5, 45.4, 12.6.

#### 1*–(2-(((6-Methoxy-4-oxo-4H-chromen-3-yl)methyl)amino)ethyl)-3-((4-methoxybenzyl)oxy)-2-methylpyridin-4(1H)-one (16e)*

Yield: 25%, yellow oil, ^1^H NMR (400 MHz, CDCl_3_) *δ* 7.83 (s, 1H), 7.53 (d, *J* = 3.1 Hz, 1H), 7.40 (d, *J* = 9.2 Hz, 1H), 7.32 − 7.27 (m, 4H), 6.85 − 6.79 (m, 2H), 6.40 (d, *J* = 7.5 Hz, 1H), 5.14 (s, 2H), 3.89 (s, 3H), 3.86 (d, *J* = 6.4 Hz, 2H), 3.77 (s, 3H), 3.58 (s, 2H), 2.84 (t, *J* = 6.4 Hz, 2H), 2.08 (s, 3H); ^13^C NMR (100 MHz, CDCl_3_) δ 177.8, 173.5, 159.5, 157.0, 153.4, 151.4, 145.8, 141.1, 139.0, 130.8, 129.7, 124.3, 124.1, 120.6, 119.7, 116.9, 113.6, 104.6, 72.6, 55.9, 55.2, 53.6, 48.5, 45.5, 12.6.

#### 1–(2-(((7-(Benzyloxy)-4-oxo-4H-chromen-3-yl)methyl)amino)ethyl)-3-((4-methoxybenzyl)oxy)-2-methylpyridin-4(1H)-one (16f)

Yield: 19%, yellow oil, ^1^H NMR (400 MHz, CDCl_3_) *δ* 8.09 (d, *J* = 8.9 Hz, 1H), 7.78 (s, 1H), 7.41 (q, *J* = 7.8 Hz, 5H), 7.30 (d, *J* = 8.6 Hz, 3H), 7.05 (dd, *J* = 8.9, 2.3 Hz, 1H), 6.90 (d, *J* = 2.3 Hz, 1H), 6.82 (d, *J* = 8.6 Hz, 2H), 6.40 (d, *J* = 7.4 Hz, 1H), 5.14 (s, 2H), 5.13 (s, 2H), 3.89 (t, *J* = 6.2 Hz, 2H), 3.76 (s, 3H), 3.57 (s, 2H), 2.85 (t, *J* = 6.2 Hz, 2H), 2.09 (s, 3H); ^13^C NMR (100 MHz, CDCl_3_) *δ* 177.3, 173.5, 163.3, 159.5, 158.2, 153.1, 145.9, 140.9, 138.9, 135.6, 130.8, 129.8, 128.8, 128.5, 127.6, 127.1, 121.2, 117.8, 117.0, 115.4, 113.6, 101.3, 72.6, 70.6, 55.2, 53.6, 48.5, 45.5, 12.7.

#### 3-((4-Methoxybenzyl)oxy)-2-methyl-1–(2-(((7-((3-methylbenzyl)oxy)-4-oxo-4H-chromen-3-yl)methyl)amino)ethyl)pyridin-4(1H)-one (16g)

Yield: 15%, yellow oil, ^1^H NMR (400 MHz, CDCl_3_) *δ* 8.08 (d, *J* = 8.9 Hz, 1H), 7.73 (s, 1H), 7.28 (t, *J* = 7.3 Hz, 3H), 7.23 (dd, *J* = 11.8, 6.2 Hz, 3H), 7.16 (d, *J* = 7.3 Hz, 1H), 7.03 (dd, *J* = 8.9, 2.2 Hz, 1H), 6.88 (d, *J* = 2.1 Hz, 1H), 6.80 (d, *J* = 8.4 Hz, 2H), 6.38 (d, *J* = 7.5 Hz, 1H), 5.13 (s, 2H), 5.09 (s, 2H), 3.83 (t, *J* = 6.3 Hz, 2H), 3.75 (s, 3H), 3.52 (s, 2H), 2.80 (t, *J* = 6.3 Hz, 2H), 2.37 (s, 3H), 2.06 (s, 3H); ^13^C NMR (100 MHz, CDCl_3_) *δ* 177.3, 173.5, 163.3, 159.4, 158.2, 153.0, 145.8, 141.0, 139.0, 138.6, 135.5, 130.8, 129.7, 129.2, 128.7, 128.3, 127.0, 124.7, 121.3, 117.8, 117.0, 115.4, 113.6, 101.3, 72.6, 70.7, 55.2, 53.7, 48.6, 45.5, 21.5, 12.7.

#### 3-((4-Methoxybenzyl)oxy)-2-methyl-1–(2-(((7-((4-methylbenzyl)oxy)-4-oxo-4H-chromen-3-yl)methyl)amino)ethyl)pyridin-4(1H)-one (16h)

Yield: 16%, yellow oil, ^1^H NMR (400 MHz, CDCl_3_) *δ* 8.08 (d, *J* = 8.9 Hz, 1H), 7.80 (s, 1H), 7.31 (t, *J* = 8.7 Hz, 5H), 7.21 (d, *J* = 7.8 Hz, 2H), 7.03 (dd, *J* = 8.9, 2.1 Hz, 1H), 6.89 (d, *J* = 2.0 Hz, 1H), 6.82 (d, *J* = 8.5 Hz, 2H), 6.40 (d, *J* = 7.4 Hz, 1H), 5.11 (d, *J* = 9.9 Hz, 4H), 3.90 (t, *J* = 6.0 Hz, 2H), 3.76 (s, 3H), 3.58 (s, 2H), 2.85 (t, *J* = 6.1 Hz, 2H), 2.36 (s, 3H), 2.08 (s, 3H); ^13^C NMR (100 MHz, CDCl_3_) *δ* 177.3, 173.4, 163.4, 159.5, 158.3, 145.8, 141.3, 139.0, 138.4, 132.4, 130.9, 129.6, 129.5, 127.8, 127.0, 117.7, 117.0, 115.5, 113.6, 101.3, 72.7, 70.6, 55.3, 53.4, 48.3, 45.3, 21.3, 12.7.

#### 1–(2-(((7-((3-Fluorobenzyl)oxy)-4-oxo-4H-chromen-3-yl)methyl)amino)ethyl)-3-((4-methoxybenzyl)oxy)-2-methylpyridin-4(1H)-one (16i)

Yield: 16%, yellow oil, ^1^H NMR (400 MHz, CDCl_3_) *δ* 8.09 (d, *J* = 8.9 Hz, 1H), 7.74 (s, 1H), 7.39 − 7.27 (m, 4H), 7.19 (d, *J* = 7.7 Hz, 1H), 7.14 (d, *J* = 9.4 Hz, 1H), 7.05 − 7.00 (m, 2H), 6.88 − 6.85 (m, 1H), 6.80 (d, *J* = 8.3 Hz, 2H), 6.38 (d, *J* = 7.4 Hz, 1H), 5.12 (s, 4H), 3.85 (t, *J* = 6.1 Hz, 2H), 3.75 (s, 3H), 3.54 (s, 2H), 2.82 (t, *J* = 6.1 Hz, 2H), 2.07 (s, 3H). ^13^C NMR (100 MHz, CDCl_3_) *δ* 177.3, 173.4, 163.0 (d, ^1^*J* = 245 Hz), 162.9, 159.4, 158.2, 153.2, 145.9, 141.0, 139.0, 138.1 (d, ^3^*J* = 8 Hz), 130.8, 130.4 (d, ^3^*J* = 242 Hz), 129.7, 127.2, 122.8 (d, ^4^*J* = 2 Hz), 121.3, 118.0, 117.0, 115.4, 115.2, 114.3 (d, ^2^*J* = 22 Hz), 113.6, 101.4, 72.6, 69.7, 55.2, 53.6, 48.5, 45.4, 12.7.

#### 1–(2-(((7-((4-Fluorobenzyl)oxy)-4-oxo-4H-chromen-3-yl)methyl)amino)ethyl)-3-((4-methoxybenzyl)oxy)-2-methylpyridin-4(1H)-one (16j)

Yield: 17%, yellow oil, ^1^H NMR (400 MHz, CDCl_3_) *δ* 8.10 (d, *J* = 8.9 Hz, 1H), 7.75 (s, 1H), 7.41 (dd, *J* = 8.5, 5.4 Hz, 2H), 7.34 − 7.27 (m, 3H), 7.12 − 7.07 (m, 2H), 7.04 (dd, *J* = 8.9, 2.4 Hz, 1H), 6.89 (d, *J* = 2.3 Hz, 1H), 6.82 (d, *J* = 8.6 Hz, 2H), 6.39 (d, *J* = 7.5 Hz, 1H), 5.15 (s, 2H), 5.10 (s, 2H), 3.86 (t, *J* = 6.3 Hz, 2H), 3.77 (s, 3H), 3.55 (s, 2H), 2.84 (t, *J* = 6.3 Hz, 2H), 2.09 (s, 3H). ^13^C NMR (100 MHz, CDCl_3_) *δ* 177.4, 173.5, 163.1, 162.8 (d, ^1^*J* = 245 Hz) 159.5, 158.3, 153.2, 145.9, 141.2, 139.1, 131.4 (d, ^4^*J* = 3 Hz), 130.9, 129.8, 129.6 (d, ^3^*J* = 9 Hz), 129.6 (d, ^3^*J* = 8 Hz), 127.2, 121.4, 118.0, 117.0, 115.8 (d, ^2^*J* = 21 Hz), 115.4, 113.7, 101.4, 72.7, 70.0, 55.3, 53.7, 48.6, 45.5, 12.7.

#### 1–(2-(((7-((3-Chlorobenzyl)oxy)-4-oxo-4H-chromen-3-yl)methyl)amino)ethyl)-3-((4-methoxybenzyl)oxy)-2-methylpyridin-4(1H)-one (16k)

Yield: 15%, yellow oil, ^1^H NMR (400 MHz, CDCl_3_) *δ* 8.10 − 8.06 (m, 1H), 7.73 (s, 1H), 7.41 (s, 1H), 7.32 − 7.27 (m, 5H), 7.24 (s, 1H), 7.02 (dd, *J* = 8.9, 2.1 Hz, 1H), 6.85 (s, 1H), 6.79 (d, *J* = 8.6 Hz, 2H), 6.38 − 6.33 (m, 1H), 5.11 (s, 2H), 5.09 (s, 2H), 3.83 (t, *J* = 6.1 Hz, 2H), 3.75 − 3.72 (m, 3H), 3.52 (s, 2H), 2.80 (t, *J* = 6.0 Hz, 2H), 2.06 (s, 3H); ^13^C NMR (100 MHz, CDCl_3_) *δ* 177.2, 173.4, 162.8, 159.4, 158.2, 153.1, 145.9, 140.9, 139.0, 137.6, 134.7, 130.8, 130.1, 129.7, 128.6, 127.5, 127.2, 125.4, 121.4, 118.0, 116.9, 115.2, 113.6, 101.4, 72.5, 69.6, 55.2, 53.6, 48.6, 45.4, 12.6.

#### 1–(2-(((7-((4-Chlorobenzyl)oxy)-4-oxo-4H-chromen-3-yl)methyl)amino)ethyl)-3-((4-methoxybenzyl)oxy)-2-methylpyridin-4(1H)-one (16l)

Yield: 16%, yellow oil, ^1^H NMR (400 MHz, CDCl_3_) *δ* 8.09 (d, *J* = 8.9 Hz, 1H), 7.73 (s, 1H), 7.37 (s, 4H), 7.31 − 7.27 (m, 3H), 7.03 (dd, *J* = 8.9, 2.2 Hz, 1H), 6.87 (d, *J* = 2.3 Hz, 1H), 6.81 (d, *J* = 8.5 Hz, 2H), 6.38 (d, *J* = 7.5 Hz, 1H), 5.12 (s, 2H), 5.10 (s, 2H), 3.84 (t, *J* = 6.3 Hz, 2H), 3.75 (s, 3H), 3.53 (s, 2H), 2.81 (t, *J* = 6.3 Hz, 2H), 2.07 (s, 3H); ^13^C NMR (100 MHz, CDCl_3_) *δ* 177.3, 173.5, 163.0, 159.5, 158.2, 153.0, 145.9, 141.0, 139.0, 134.3, 134.1, 130.8, 129.7, 129.0, 128.9, 127.2, 121.4, 118.0, 116.9, 115.2, 113.6, 101.3, 72.6, 69.8, 55.2, 53.7, 48.6, 45.5, 12.7.

### Determination of pK_a_ and Fe^3+^ affinity by spectrophotometry method

The experimental procedures of the pKa and Fe^3+^ affinity assay were performed according to our reported methods^20^. For a detailed experiment protocol, please refer to the supporting information.

### Monoamine oxidase enzyme assay and kinetic studies

The reagents and enzymes (Human MAO-B and Human MAO-A) applied in this experiment were purchased from Sigma-Aldrich. According to the manuals, all components (substrate solution and enzyme solution) are diluted and placed aside for use. The detailed process has been mentioned in our early work^34^.

To further examine the interaction mode of compound **17d**, the type of enzyme inhibition was determined by Michealis–Menten kinetic experiments. The catalytic rates of MAO‐B enzyme were measured at six different concentrations of substrate *p*‐tyramine (0.05, 0.1, 0.25, 0.5, 1.0, and 1.25 mM) in the absence and in the three different concentrations (33.3, 100, and 300 nM) of compound **17d**. The corresponding dose‐response curves and the nonlinear/linear regression analysis were performed using GraphPad Prism version 6 software (Graphpad Software Inc., La Jolla, CA).

### PAMPA-BBB assay

PAMPA was used to predict the BBB permeability of the screened compound. The detailed process has been mentioned in our early work^32^^,^^33^.

### Cytotoxicity test

Neural pheochromocytoma-derived PC-12 cells at the log phase of their growth cycle (8 × 10^4^ cells/ml) were added to each well (100 μl/well) and incubated for 24 h at 37 °C in a humidified atmosphere of 5% CO_2_. Then three replicates at various concentrations of the samples (3.13, 6.25, 12.5, 25, and 50 μM) were added to each well (100 μl/well). After incubating for 24 h, 20 μl of MTT solution (5 mg/ml/well) was added to each culture medium, which was then incubated for another 4 h. Then DMSO was added to each well (150 μl/well). After 10 min at room temperature, the OD values of each well were measured on a Microplate Reader (BioTek ELx800, Winooski, VT) at the wavelength of 490 nm.

### Molecular modelling studies

According to our previous methods^20^, docking simulation was detailed in the supporting information, using crystal structures of MAO-B in complex with safinamide (PDB code 2V5Z)^35^ as the template. For a detailed experiment protocol, please refer to the supporting information.

### Morris water maze test

Adult female ICR mice (weight 20–25 g) were obtained from the Zhejiang Academy of Medical Sciences (Hangzhou, China). Scopolamine hydrobromide and Pargyline were purchased from Aladdin Reagents. Memantine was purchased from Energy Chemical. All experiments were approved by the Laboratory Animals Ethical Committee of the Zhejiang University of Technology (No. 20211210103). In this test, mice were prepared as clarified and clear injections, consisting of 20% (2-Hydroxypropyl)-*β*-cyclodextrin, 10% DMSO, and PBS. The mice were randomly quartered into five groups: (i) control group (PBS of 20% (2-Hydroxypropyl)-*β*-cyclodextrin), (ii) scopolamine hydrobromide model group (15 mg/kg), (iii) pargyline group (15 mg/kg) + 15 mg/kg scopolamine hydrobromide, (iv) memantine group (15 mg/kg) + 15 mg/kg scopolamine hydrobromide, and (v) compound **17d** group (15 mg/kg) + 15 mg/kg scopolamine hydrobromide. Mice were administered at a frequency of once a day over 15 d with a fixed dosing schedule. The body weight of mice was measured during the experiment.

After eliminating environmental stress, the behavioural test was performed over the next five days. The mice were trained to find the platform (10 cm diameter) in the circular light-avoidance pool (120 cm diameter and 60 cm height) filled with water (40 cm, depth), which was described in detail in our previous study^34^. All mice received at least one training session daily in four quadrants for four consecutive days before the probe trial of finding the platform within 120 s. Recordings were made with SMART version 3.0 (Panlab, Spain) and processed by Graphpad Prism version 6 software (Graphpad Software Inc., La Jolla, CA, USA).

### Pharmacokinetic study

Rats were purchased from the Zhejiang Academy of Medical Sciences (Hangzhou, China). All rats were kept strictly following the requirements of experimental animal operation instructions, kept at a temperature (22 ± 2 °C), day/night cycle every 12 h in a constant environment. 12 h before the experiment, the rats were fasted but free to drink. For each rat, retro-orbital blood samples (0.2 ml) were obtained into EDTA soaked polythene tubes before drug administration and at time points of 0.083, 0.25, 0.50, 0.75, 1.0, 2.0, 4.0, 6.0, 8.0, 10, 12, and 24 h post-dosing. The collected blood samples were immediately centrifuged at 3500 rpm for 10 min at room temperature to separate plasma, and stored in a −80 °C refrigerator before use. For brain tissue distribution investigation, rats were sacrificed at *C*_max_ time point after i.g. administration (15 mg/kg) of **17d**. The tissue was weighed and homogenised under ice bath conditions and was collected for further processing.

Biological samples were performed with ACQUITY UPLC^®^ H Class system, which was coupled to an Xevo TQ-S micro triple quadrupole mass spectrometer (Waters, Milford, MA, USA) with an ESI source and MassLynx^TM^ Workstation software version 4.2 (Waters, Milford, MA, USA). We used a BEH Shield RP C18 column (100 × 2.1 mm, 1.7 µm, Waters, Milford, MA, USA) for chromatographic separation, and the column temperature is kept at 30 °C. The mobile phase was composed of 30%, 0.2% formic acid aqueous solution (A) and 70%, 0.3% formic acid methanol solution (B) and is delivered at a flow rate of 0.2 ml/min. The temperature of the autosampler is set to 30 °C. The sample injection volume is 5 µl. Pharmacokinetic parameters of the analytes were calculated using the pharmacokinetic software DAS version 2.0 (Bontz Inc., Beijing, China). All data were expressed as mean ± SD.

## References

[1] Alzheimer's Association. Alzheimer’s disease facts and figures. Alzheimer’s Dement. 2021;17:327–406. 33756057 10.1002/alz.12328

[2] Srivastava S, Ahmad R, Khare SK. Alzheimer’s disease and its treatment by different approaches: a review. Eur J Med Chem. 2021;216:113320. 33652356 10.1016/j.ejmech.2021.113320

[3] Abeysinghe A, Deshapriya R, Udawatte C. Alzheimer’s disease; a review of the pathophysiological basis and therapeutic interventions. Life Sci. 2020;256:117996. 32585249 10.1016/j.lfs.2020.117996

[4] Kepp KP. Bioinorganic chemistry of Alzheimer’s disease. Chem Rev. 2012;112(10):5193–5239. 22793492 10.1021/cr300009x

[5] Telling ND, Everett J, Collingwood JF, Dobson J, van der Laan G, Gallagher JJ, Wang J, Hitchcock AP. Iron biochemistry is correlated with amyloid plaque morphology in an established mouse model of Alzheimer’s disease. Cell Chem Biol. 2017;24(10):1205–1215 e3. 28890316 10.1016/j.chembiol.2017.07.014

[6] Savelieff MG, Nam G, Kang J, Lee HJ, Lee M, Lim MH. Development of multifunctional molecules as potential therapeutic candidates for Alzheimer’s disease, Parkinson’s disease, and amyotrophic lateral sclerosis in the last decade. Chem Rev. 2019;119(2):1221–1322. 30095897 10.1021/acs.chemrev.8b00138

[7] Bai R, Guo J, Ye XY, Xie Y, Xie T. Oxidative stress: the core pathogenesis and mechanism of Alzheimer’s disease. Ageing Res Rev. 2022;77:101619. 35395415 10.1016/j.arr.2022.101619

[8] Lane DJR, Ayton S, Bush AI. Iron and Alzheimer’s disease: an update on emerging mechanisms. J Alzheimers Dis. 2018;64(s1):S379–S395. 29865061 10.3233/JAD-179944

[9] Mesiti F, Chavarria D, Gaspar A, Alcaro S, Borges F. The chemistry toolbox of multitarget-directed ligands for Alzheimer’s disease. Eur J Med Chem. 2019;181:111572. 31404859 10.1016/j.ejmech.2019.111572

[10] Jiang X, Zhou T, Bai R, Xie Y. Hydroxypyridinone-based iron chelators with broad-ranging biological activities. J Med Chem. 2020;63(23):14470–14501. 33023291 10.1021/acs.jmedchem.0c01480

[11] Cong L, Dong X, Wang Y, Deng Y, Li B, Dai R. On the role of synthesized hydroxylated chalcones as dual functional amyloid-beta aggregation and ferroptosis inhibitors for potential treatment of Alzheimer’s disease. Eur J Med Chem. 2019;166:11–21. 30684867 10.1016/j.ejmech.2019.01.039

[12] Tripathi AC, Upadhyay S, Paliwal S, Saraf SK. Privileged scaffolds as MAO inhibitors: retrospect and prospects. Eur J Med Chem. 2018;145:445–497. 29335210 10.1016/j.ejmech.2018.01.003

[13] Manzoor S, Hoda N. A comprehensive review of monoamine oxidase inhibitors as anti-Alzheimer’s disease agents: a review. Eur J Med Chem. 2020;206:112787. 32942081 10.1016/j.ejmech.2020.112787

[14] Zhou J, Jiang X, He S, Jiang H, Feng F, Liu W, Qu W, Sun H. Rational design of multitarget-directed ligands: strategies and emerging paradigms. J Med Chem. 2019;62(20):8881–8914. 31082225 10.1021/acs.jmedchem.9b00017

[15] Wang T, Liu XH, Guan J, Ge S, Wu MB, Lin JP, Yang LR. Advancement of multi-target drug discoveries and promising applications in the field of Alzheimer’s disease. Eur J Med Chem. 2019;169:200–223. 30884327 10.1016/j.ejmech.2019.02.076

[16] Carradori S, Silvestri R. New frontiers in selective human MAO-B inhibitors. J Med Chem. 2015;58(17):6717–6732. 25915162 10.1021/jm501690r

[17] Liu W, Lang M, Youdim MBH, Amit T, Sun Y, Zhang Z, Wang Y, Weinreb O. Design, synthesis and evaluation of novel dual monoamine-cholinesterase inhibitors as potential treatment for Alzheimer’s disease. Neuropharmacology. 2016;109:376–385. 27318273 10.1016/j.neuropharm.2016.06.013

[18] Mechlovich D, Amit T, Mandel SA, Bar-Am O, Bloch K, Vardi P, Youdim MBH. The novel multifunctional, iron-chelating drugs M30 and HLA20 protect pancreatic beta-cell lines from oxidative stress damage. J Pharmacol Exp Ther. 2010;333(3):874–882. 20237072 10.1124/jpet.109.164269

[19] Esteban G, Allan J, Samadi A, Mattevi A, Unzeta M, Marco-Contelles J, Binda C, Ramsay RR. Kinetic and structural analysis of the irreversible inhibition of human monoamine oxidases by ASS234, a multi-target compound designed for use in Alzheimer’s disease. Biochim Biophys Acta. 2014;1844(6):1104–1110. 24642166 10.1016/j.bbapap.2014.03.006

[20] Zhang C, Yang K, Yu S, Su J, Yuan S, Han J, Chen Y, Gu J, Zhou T, Bai R, et al. Design, synthesis and biological evaluation of hydroxypyridinone-coumarin hybrids as multimodal monoamine oxidase B inhibitors and iron chelates against Alzheimer’s disease. Eur J Med Chem. 2019; 180:367–382. 31325784 10.1016/j.ejmech.2019.07.031

[21] Pachón-Angona I, Refouvelet B, Andrýs R, Martin H, Luzet V, Iriepa I, Moraleda I, Diez-Iriepa D, Oset-Gasque M-J, Marco-Contelles J, et al. Donepezil + chromone + melatonin hybrids as promising agents for Alzheimer’s disease therapy. J Enzyme Inhib Med Chem. 2019;34(1):479–489. 30712420 10.1080/14756366.2018.1545766 PMC6366423

[22] Jiang X, Guo J, Zhang C, Gu J, Zhou T, Bai R, Xie Y. Discovery of benzamide-hydroxypyridinone hybrids as potent multi-targeting agents for the treatment of Alzheimer’s disease. J Enzyme Inhib Med Chem. 2021;36(1):2045–2054. 34607518 10.1080/14756366.2021.1978081 PMC8510601

[23] Reis J, Cagide F, Chavarria D, Silva T, Fernandes C, Gaspar A, Uriarte E, Remião F, Alcaro S, Ortuso F, et al. Discovery of new chemical entities for old targets: insights on the lead optimization of chromone-based monoamine oxidase B (MAO-B) inhibitors. J Med Chem. 2016;59(12):5879–5893. 27244485 10.1021/acs.jmedchem.6b00527

[24] Fonseca A, Reis J, Silva T, Matos MJ, Bagetta D, Ortuso F, Alcaro S, Uriarte E, Borges F. Coumarin versus chromone monoamine oxidase B inhibitors: quo vadis? J Med Chem. 2017;60(16):7206–7212. 28753307 10.1021/acs.jmedchem.7b00918

[25] Reis J, Gaspar A, Milhazes N, Borges F. Chromone as a privileged scaffold in drug discovery: recent advances. J Med Chem. 2017;60(19):7941–7957. 28537720 10.1021/acs.jmedchem.6b01720

[26] Mi Z, Gan B, Yu S, Guo J, Zhang C, Jiang X, Zhou T, Su J, Bai R, Xie Y. Dual-target anti-Alzheimer’s disease agents with both iron ion chelating and monoamine oxidase-B inhibitory activity. J Enzyme Inhib Med Chem. 2019;34(1):1489–1497. 31416364 10.1080/14756366.2019.1634703 PMC6713216

[27] Rullo M, Catto M, Carrieri A, de Candia M, Altomare CD, Pisani L. Chasing ChEs-MAO B multi-targeting 4-aminomethyl-7-benzyloxy-2-chromen-2-ones. Molecules. 2019;24(24):4507. 31835376 10.3390/molecules24244507 PMC6943664

[28] Zeng H, Wu X. Alzheimer’s disease drug development based on computer-aided drug design. Eur J Med Chem. 2016; 121:851–863. 26415837 10.1016/j.ejmech.2015.08.039

[29] Gaspar A, Silva T, Yanez M, Vina D, Orallo F, Ortuso F, Uriarte E, Alcaro S, Borges F. Chromone, a privileged scaffold for the development of monoamine oxidase inhibitors. J Med Chem. 2011;54(14):5165–5173.] 21696156 10.1021/jm2004267

[30] Masaldan S, Bush AI, Devos D, Rolland AS, Moreau C. Striking while the iron is hot: iron metabolism and ferroptosis in neurodegeneration. Free Radic Biol Med. 2019;133:221–233. 30266679 10.1016/j.freeradbiomed.2018.09.033

[31] Xie Y-Y, Lu Z, Kong X-L, Zhou T, Bansal S, Hider R. Systematic comparison of the mono-, dimethyl- and trimethyl 3-hydroxy-4(1H)-pyridones – attempted optimization of the orally active iron chelator, deferiprone. Eur J Med Chem. 2016;115:132–140. 27014847 10.1016/j.ejmech.2016.03.014

[32] Guo J, Zhang Y, Zhang C, Yao C, Zhang J, Jiang X, Zhong Z, Ge J, Zhou T, Bai R, et al. N-propargylamine-hydroxypyridinone hybrids as multitarget agents for the treatment of Alzheimer’s disease. Bioorg Chem. 2021;113:105013. 34062405 10.1016/j.bioorg.2021.105013

[33] Di L, Kerns EH, Fan K, McConnell OJ, Carter GT. High throughput artificial membrane permeability assay for blood–brain barrier. Eur J Med Chem. 2003;38(3):223–232. 12667689 10.1016/s0223-5234(03)00012-6

[34] Guo J, Mi Z, Jiang X, Zhang C, Guo Z, Li L, Gu J, Zhou T, Bai R, Xie Y. Design, synthesis and biological evaluation of potential anti-AD hybrids with monoamine oxidase B inhibitory and iron-chelating effects. Bioorg Chem. 2021;108:104564. 33353806 10.1016/j.bioorg.2020.104564

[35] Binda C, Wang J, Pisani L, Caccia C, Carotti A, Salvati P, Edmondson DE, Mattevi A. Structures of human monoamine oxidase B complexes with selective noncovalent inhibitors: safinamide and coumarin analogs. J Med Chem. 2007;50(23):5848–5852. 17915852 10.1021/jm070677y

